# The cytomegalovirus protein US31 induces inflammation through mono-macrophages in systemic lupus erythematosus by promoting NF-κB2 activation

**DOI:** 10.1038/s41419-017-0122-4

**Published:** 2018-01-24

**Authors:** Gangqiang Guo, Sisi Ye, Shangdan Xie, Lele Ye, Cong Lin, Min Yang, Xinyu Shi, Fangyan Wang, Baoqing Li, Ming Li, Chaosheng Chen, Lifang Zhang, Huidi Zhang, Xiangyang Xue

**Affiliations:** 1 Department of Microbiology and Immunology, Institute of molecular virology and immunology, Institute of Tropical Medicine, Wenzhou Medical University, Wenzhou, China; 2 Second Clinical College, Wenzhou Medical University, Wenzhou, China; 3 Department of Pathophysiology, Wenzhou Medical University, Wenzhou, China; 4 Department of Laboratory Medicine, Second Affiliated Hospital & Yuying Children’s Hospital, Wenzhou Medical University, Wenzhou, China; 5 Cardiac regeneration research institute, Wenzhou Medical University, Wenzhou, China; 6 Department of Nephrology, First Affiliated Hospital, Wenzhou Medical University, Wenzhou, China

## Abstract

It has been hypothesized that human cytomegalovirus (HCMV) infection, especially in monocyte and CD34 (+) myeloid cells, acts as a important regulator of immune system to promote inflammation in multiple autoimmune diseases. The aim of this study was to elucidate the HCMV gene expression profiles in the peripheral blood mononuclear cells (PBMCs) of SLE patients and demonstrate the effect and mechanism of viral gene associated with SLE in mono-macrophages functions. Using two RNA-Seq techniques in combination with RT-PCR, 11 viral genes mainly associated with latent HCMV infection were identified in the PBMCs of SLE patients. Among these viral genes, US31 with previously unknown function was highly expressed in the PBMCs of SLE patients compared to healthy controls. Analysis of function indicated that US31 expression could induce inflammation in monocyte and macrophage and stimulate macrophage differentiation toward an M1 macrophage phenotype. Screening via protein chips in combination with bioinformatic analysis and consequent detection of mono-macrophages function indicates that the direct interaction between US31 and NF-κB2 contributed the NF-kB2 activation. Consequent analysis indicated US31 directly interacted with NF-κB2, contribute to the polyubiquitination of the phosphorylated p100 and consequent activation of NF-κB2. Taken together, our data uncovered a previously unknown role of the HCMV protein US31 in inducing NF-κB-mediated mono-macrophage inflammation in the pathogenesis and development of SLE. Our findings provide a foundation for the continued investigation of novel therapeutic targets for SLE patients.

## Introduction

Increasing evidence suggests that human cytomegalovirus (HCMV), which commonly infects human populations, constitutes an important trigger of SLE and can further aggravate disease progression^1–3^. In turn, systemic lupus erythematosus (SLE) aggravation also promotes HCMV infection^4^, resulting in a vicious cycle. However, there is currently very little information available regarding the mechenisms after HCMV infection with the immunological function and SLE morbidity.

It has been demonstrated that the abnormalities in monocyte function can impair immune homeostasis, thereby activate autoimmune responses and result in SLE-associated immune-mediated damage^5,6^. Whereas, monocytes and consequent differentiation are verified as playing a decisive and crucial role in the systemic spread of HCMV as well as establishment of effective and persistent infection ^6^. It is, therefore, possible that phenotypic and functional abnormalities caused by HCMV infection of monocytes play a crucial role in the occurrence and development of SLE. However, in the current literature, only a few studies have reported the effects of HCMV infection on monocyte function^5,7^.

HCMV encodes 252 open reading frames in the 230-kb genome and was believed to encode approximately 180 proteins^8^. HCMV infection involves significant changes in gene expression in different cell types at different stages of infection. However, a global investigation of differentially viral gene expression in HCMV-infected immune cells in SLE patients and their effects on cell function have never been performed. So, in this study, we utilized high-throughput RNA-Seq in combination with RT-PCR to explore the profile of HCMV gene expression in the peripheral blood mononuclear cells (PBMCs) of SLE patients. The effects and mechanism of SLE-associated US31 gene expression on monocyte immunological function were also investigated. The results of this study will provide a foundation for new concepts regarding SLE pathogenesis as well as novel strategies to prevent and treat this disease.

## Results

### Global profile of HCMV gene expression in PBMCs of SLE patients

**Fig. 1:**
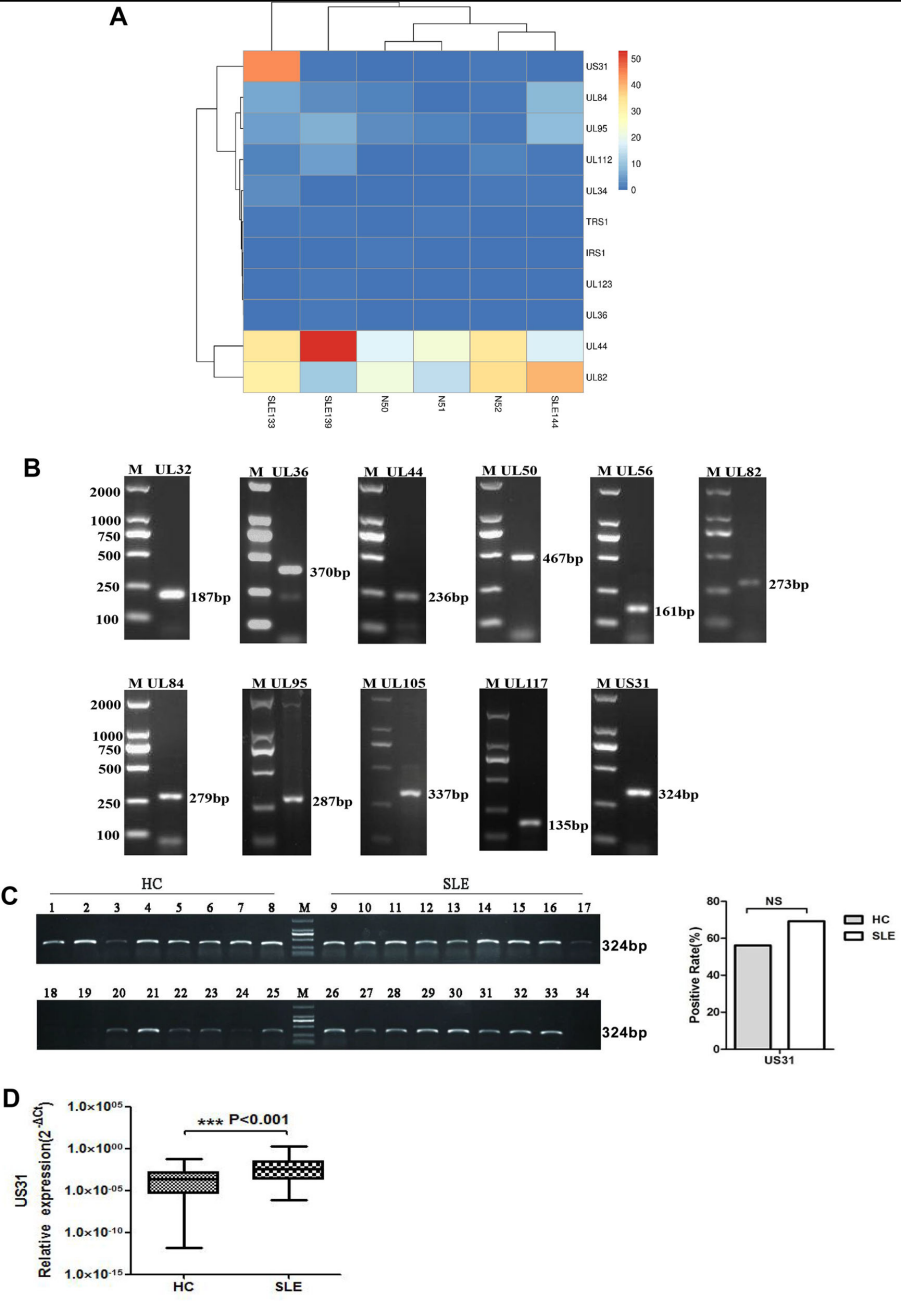
Detection of global HCMV gene expression in SLE patients and demonstration that US31 is an SLE-associated HCMV gene Transcriptome sequencing was performed to detect HCMV gene expression in the PBMCs from three SLE patients and three normal controls. **a** Cluster analysis of HCMV transcripts detected along with a bar graph representing the HCMV gene expression for each positive sample using rRNA removal + strand specific-seq. **b** PCR verification of SLE-associated HCMV genes. The figure does not show the electrophoresis image of negatively detected genes. The samples analyzed in panel B are from four additional samples of SLE PBMCs. They are from unique donors, and do not overlap with those used for Fig. 1a and Figure S1A. M: Marker. **c** Representative image of PCR electrophoresis for US31 expression in PBMCs isolated from SLE patients and healthy controls (HC) (Left panel, only several results are shown). Quantitation of the positivity rate of US31 expression in PBMCs from SLE patients and healthy controls (Right panel). A chi-square test was used to compare the differences in positivity rates between both groups. *NS* no statistical significance. **d** Box and whisker plots of the qPCR-quantitated US31 expression in PBMCs from SLE patients and healthy controls. A non-parametric Mann–Whitney *U* test was used to compare the differences in expression levels between SLE patients and healthy controls. The top and bottom of the boxes represent the 75th and 25th percentiles, respectively, whereas the band in the middle of the boxes represents the 50th percentile (median). The error bars represent the minimum to maximum values. ***P < 0.001

To analyze the profile of HCMV gene expression in the PBMCs of SLE patients, we randomly selected PBMCs from three SLE patients for whole transcriptome sequencing and mRNA sequencing. Our data showed that 18 viral genes (UL29, UL32, UL34, UL36, UL37, UL44, UL50, UL56, UL82, UL84, UL95, UL105, UL112, UL117, UL123, US3, US31, and TRS1) were detected in the PBMCs of at least one of the three SLE patients. Of these,seven (UL34, UL44, UL82, UL84, UL95, UL112, and TRS1) were detected by both sequencing methods. Alternatively, UL36, UL123, and US31 were only detected during whole transcriptome sequencing, whereas UL29, UL32, UL37, UL50, UL56, UL105, UL117, and US3 were only detected during mRNA sequencing, respectively. Among 18 HCMV genes, seven (UL34, UL44, UL50, UL82, UL84, UL95, and UL11) were detected in the PBMCs of all three SLE patients, with four additional genes (US31, UL32, UL105, and TRS1) being detected from two SLE patients and seven (UL29, UL36, UL37, UL56, UL117, UL123, and US3) from one SLE patients. Moreover, 17 viral genes (UL29, UL32, UL34, UL37, UL44, UL50, UL56, UL82, UL84, UL95, UL105, UL112, UL117, UL123, US31, TRS1, US3) were also detected in the PBMCs of at least one of the three healthy controls whereasUL69, and IRS1 were only detected in healthy control PBMCs (Fig. 1a and Figure S1A). Cluster analysis indicated that UL44, UL82, UL84, UL95, and US31 had the highest expression levels (Fig. 1a and Figure S1A). PCR was also performed for 11 HCMV genes (UL32, UL36, UL44, UL50, UL56, UL82, UL84, UL95, UL105, UL117, and US31) using normalized RNA quantities from another four SLE PBMC samples, further verifying their expression (Table S1, Fig. 1b).

### US31 is an SLE-associated HCMV gene

Of the three HCMV genes detected only in the PBMCs of SLE patients during whole transcriptome but not by mRNA sequencing, UL36 and UL123 were confirmed as protein coding. US31 belongs to the US1 family and is highly expressed in M1 cells, M2 cells, and dendritic cells (DCs) derived from monocytes in HCMV-infected patients^9^. However, its function remains largely unknown^8^. In the present study, US31 gene expression was similarly detected in THP-1 cells infected with clinical HCMV strains (Figure S1B). Moreover, we also used RT-PCR to evaluate the expression of US31 in PBMCs from 73 SLE patients and 75 healthy controls. There was no significantly different in the positive rate for US31 detection in SLE patients (71.23% [52/73]) comparing to healthy controls (61.33% [46/75]) (Fig. 1c). but the level of US31 expression was significantly higher in SLE patients than in healthy controls (*P* < 0.001) (Fig. 1d), which was similar the detection of anti-HCMV antibody IgG titer whereas IgM titer showed no difference (Figure S1C and S1D). In contrast, anti-US31 antibody was significantly lower in SLE patients than in healthy controls (Figure S1E). We further explored the relationship between US31 expression in PBMCs and patient clinical features. Red blood cell numbers (*P* = 0.043), complement C3 levels (*P* = 0.028), and complement C4 levels (*P* = 0.022) were significantly lower in US31-positive than US31-negative SLE patients (Table S2). The numbers of white blood cells, neutrophils, lymphocytes, and monocytes, 24-h proteinuria levels, Systemic Lupus Erythematosus Disease Activity Index (SLEDAI) score, anti-ds-DNA antibodies, U1-nuclear ribonucleoprotein (anti-U1RNP) antibodies, anti-histone antibodies, anti-Ro (anti-SS-A) antibodies, anti-La (anti-SS-B) antibodies, and other clinical indicators showed no significantly different in the US31-positive and US31-nagtive SLE patients.

### US31 is a highly conserved, protein-coding viral gene

**Fig. 2:**
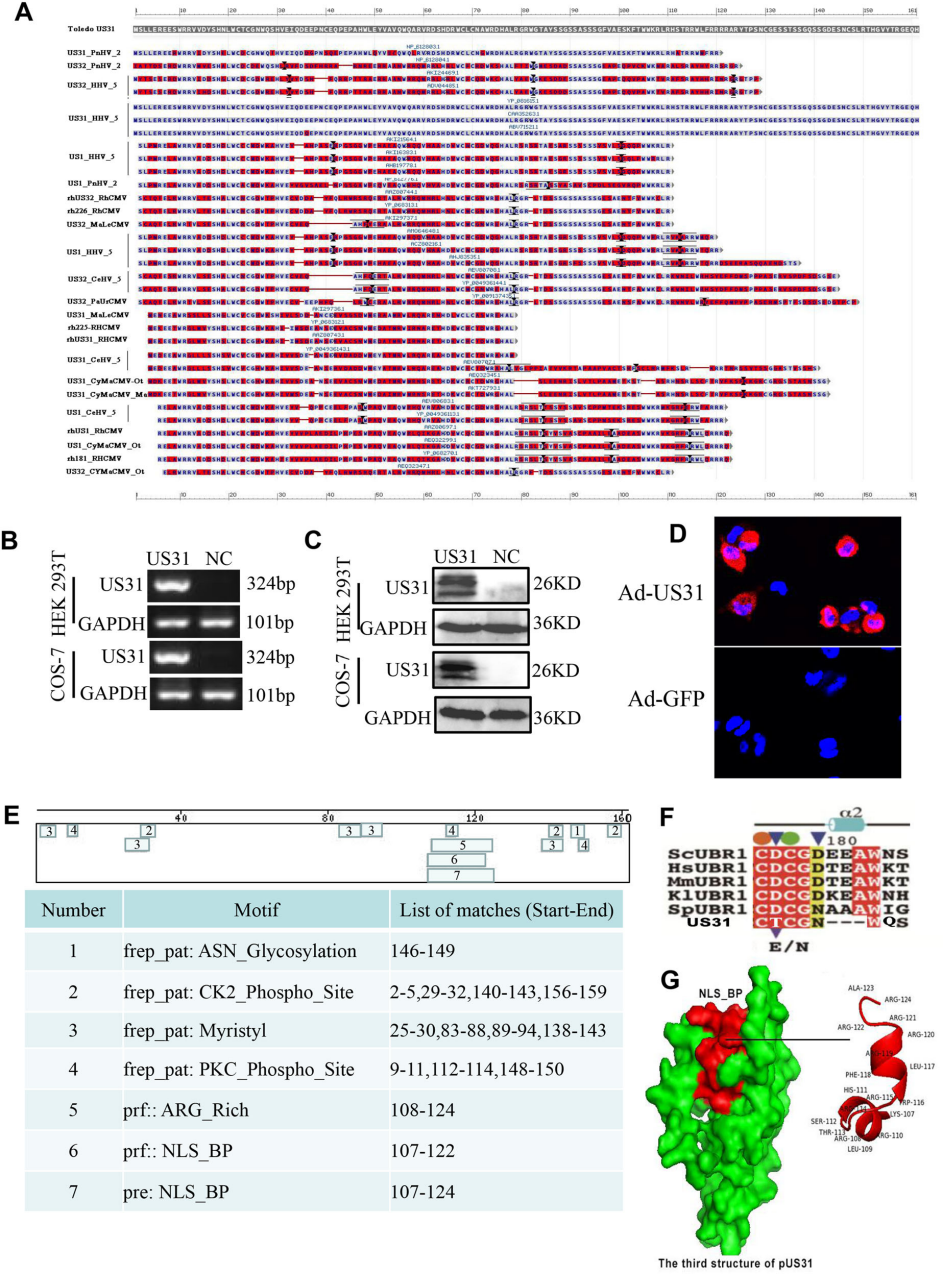
US31 gene expression **a** NCBI Blast homology analysis of the US31 gene using the Toledo strain as a reference sequence. Blue represents identity with the reference strain whereas red represents differences. PnHV_2: Panine herpesvirus 2; HHV_5: Human herpesvirus 5; Rh_CMV: Rhesus Cytomegalovirus; MaLe_CMV: Mandrillus leucophaeus cytomegalovirus; CeHV_5: Cercopithecine herpesvirus 5; PaUrCMV: *Papio ursinus* cytomegalovirus; CeHV: Cercopithecine herpesvirus 5; CyMaCMV_ot/ma: Cynomolgus macaque cytomegalovirus strain Ottawa/Mauritius. **b** mRNA detection of the US31 gene in HEK293T and COS-7 cells after infection/ transfection with Ad-US31 or Ad-GFP (NC, control) or pcDNA3.1/US31 vector (Figures do not show) for 24 h. Expression was normalized to that of *GAPDH*. **c** Western blot analysis of US31 protein expression in HEK293T and COS-7 cells 24 h after infection/ transfection with Ad-US31 or Ad-GFP (NC) or pcDNA3.1/US31 (Figures do not show). **d** Analysis of US31 expression in Ad-US31- or Ad-GFP (control)-infected THP-1-derived macrophages (48 h). Red staining indicates US31 expression (performed using a rabbit antibody against US31); blue staining indicates nuclei (DAPI). **e** Output of Expasy functional analysis of US31. **f** Sequence alignment of the UBR domain in Ubr1 E3 ligases from different organisms (*Sc, Saccharomyces cerevisiae; Hs, Homo sapiens; Mm, Mus musculus; Kl, Kluyveromyces lactis; Sp, Schizosaccharomyces pombe*). Shading indicates residues that are identical (red) or highly conserved (yellow) in all sequences. Filled circles indicate residues that coordinate the zinc ions (orange for Zn1, green for Zn2). Key determinants for binding of the N-terminal residue of the substrate are marked with blue triangles and critical residues identified by previous genetic screening are indicated with purple triangles below the sequence^39^. **g** Predicted 3-dimensional protein structure of US31. The red area represents the nuclear localization signal sequence

US31 sequence exhibits a high conservation in HCMV clinical isolates and the experimental strains. The homology of US31 in protein sequences is 99–100% in different human CMV strains, whereas its homology with viral genes from the other genus is 33–48%. Additionally, homology analysis showed that the US31 gene and the US32 and US31 genes from *Mandrillus leucophaeus* cytomegalovirus, cynomolgus macaque cytomegalovirus strain Ottawa, and cercopithecine herpesvirus 5 all showed high levels of homology (Fig. 2a). To verify that US31 is protein coding, we cloned the US31 coding region, C-terminally tagged with 3 × Flag tag, into an adenoviral vector (Ad-US31) or pcDNA3.1/US31 vector to infect or transfect HEK293T and COS-7 cells. We used an antibody against the 3 × Flag tag as well as a self-made US31-specific antibody to detect US31 expression. We found that US31 protein is expressed in Ad-US31-transfected cells (Fig. 2b) and appears to be synthesized as three species with molecular masses of 22, 24, and 26 kDa (predicted size is 20 kDa) in these cell types (Fig. 2c). Moreover, immunofluorescence microscopy also showed US31 protein expression (Fig. 2d). US31 sequence analysis showed that it contains multiple modification sites (Fig. 2e), which maybe the reason with higher molecular masses compared the predicted size. Structural analysis of the US31 gene suggests that US31 might constitute an E3 ubiquitin-protein ligase (ubr1), two zinc ion domain (Fig. 2f) and two possible nuclear localization signals (Fig. 2f, g), which indicated that US31 may participate in target protein degradation and highlight potential transcription factor function.

### US31 induces inflammation and stimulates THP-1/THP-1-derived macrophage differentiation toward an M1 macrophage phenotype

**Fig. 3:**
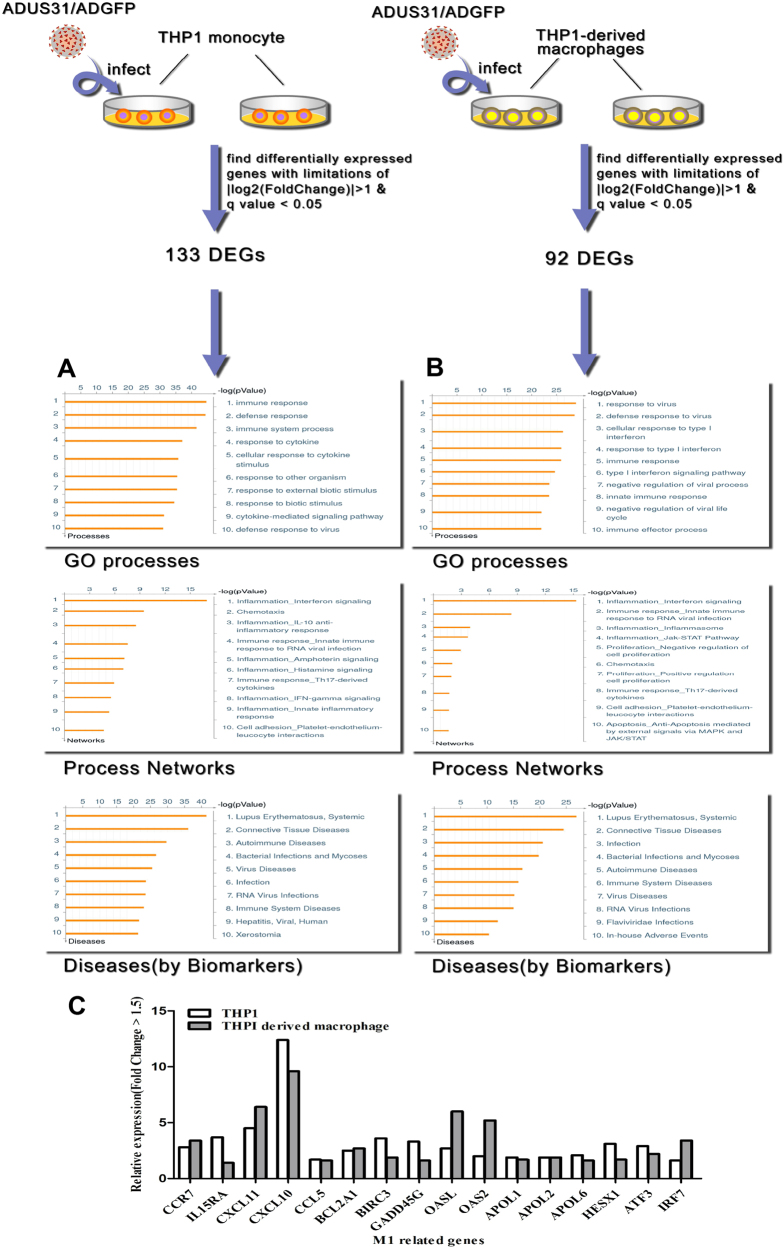
Effect of US31 expression on cellular function of monocytes/macrophages The most significant GO Process, Process Network, and Disease (by Biomarkers) terms for the differentially expressed genes detected in our RNA-Seq analysis of Ad-US31-infected THP-1 cells **a** and THP-1-derived macrophages **b**. The terms were filtered in accordance with *P* < 0.05 and FDR < 0.05. The top 10 significantly enriched terms are shown (software from Thomson Reuters Corporation was used to analyze differentially expressed genes). **c** A total of 16 of the M1-associated genes are upregulated in THP1 and THP-1-derived macrophages following infection with Ad-US31 (Fold change > 1.5 except IL15RA is 1.4 in THP-1-derived macrophage)

Transcriptome sequencing was used to explore the effects of US31 expression on the function of THP-1 monocytes and THP-1-derived macrophages. There were 133 differentially expressed genes in US31-expressed THP-1 cell (Table S3) and 92 differentially expressed genes in THP-1-derived macrophages (Table S4) comparing to control groups. A subset of 18 randomly genes were chosen to verify the RNA-Seq data and showed highly concordant (*r *= 0.63, Pearson correlation) (Figure S2A, Fig. S2b, c). A similar qRT-PCR analysis was used to confirm the quality and robustness of the RNA-Seq data of the transfected THP-1 cells (Figure S2D, Figure S2E). The consequent bioinformatics analysis indicated that US31 expression induces the inflammatory response in both THP-1 cells (GO:0006954, Table S5) and THP-1-derived macrophages(GO:0006954; Table S6). Especially, several hallmark changes were found in known NF-κB regulators and signaling molecules including an upregulation of interleukin (IL)-1β, RelB, TNF, ICAM1, IL-8, and CCL2 in US31-expressed THP-1 cells and THP-1-derived macrophages (Table S7). Disease (by Biomarkers) analysis of the differentially expressed genes in both THP-1 cells and THP-1-derived macrophages after transfection showed that US31 expression is related to the development of autoimmune diseases such as SLE (Figs. 3a, b).

Chan et al.^7^ showed that HCMV infection reprograms monocyte differentiation toward the M1 macrophage phenotype. Subsequently, Mukherjee et al.^10^ also found that non-classical monocytes from SLE patients display inflammatory features, further supporting the M1 phenotype. To further investigate the effects of Ad-US31 infection on THP-1/THP-1-derived macrophage differentiation, we compared the Ad-US31-infected cell transcriptomes to the known immunophenotypic profiles of M1 and M2 phenotype described by Martinez et al.^11^. Supplementary Tables S8-S11 show the lists of genes found to be strictly associated with M1 or M2 monocyte/macrophage polarization. Compared to controls, 24 (51%) and 23 (52%) M1-associated genes were upregulated in US31-expressed THP-1 cells and THP-1-derived macrophages, respectively (Table S8 and S10). And a total of 16 of them are simultaneously upregulated in THP1 and THP-1-derived macrophages following infection with Ad-US31 (Fig. 3c). In contrast, of the genes typically associated with the anti-inflammatory M2 phenotype, only 10 (28%) and 9 (27%) were upregulated in US31-expressed THP-1 cells and THP-1-derived macrophages, respectively (Table S9 and S11). These data show a clear transcriptional preference toward an M1 macrophage activation phenotype following Ad-US31 infection in both THP-1 cells and THP-1-derived macrophages.

### NF-κB2 is responsible for the immune function of US31 in mono-macrophages

**Fig. 4:**
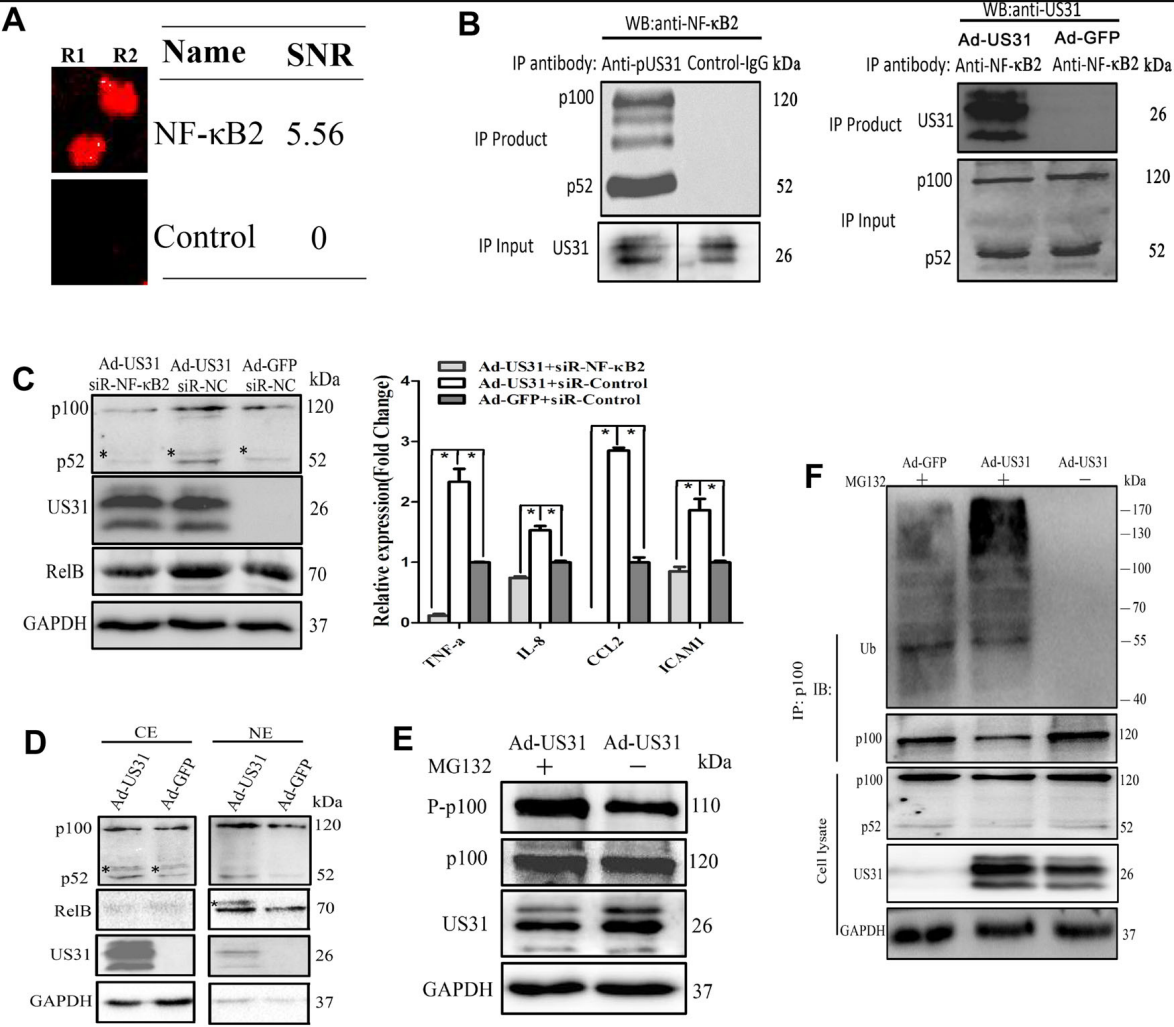
US31 interacts with NF-κB protein signaling **a** The interaction between US31 and NF-κB2 was screened by a human proteome microarray against pure US31 protein. The signal-to-noise ratio (SNR) indicated the average ratio of signal to noise calculated from two duplicate spots. R1: repetition 1; R2: repetition 1. **b** US31 forms a complex with NF-κB2 in Ad-US31-infected THP-1-derived macrophages (48 h) that can be detected following both US31/Flag and NF-κB2 immunoprecipitation (IP) and subsequent western blot analysis with the other antibody as shown (Figures do not show the IP results of anti-Flag). In each experiment, lysates incubated with control IgG (Antibody purified from control group immune serum) served as negative controls. The immunoprecipitation assays are representative of 3 independent experiments. **c** Inhibition of US31 in cell inflammation was verified by siRNA-NF-κB2. THP1 derived macrophages at 48 h post infection with Ad-US31 (or Ad-GFP) and NF-κB2 siRNA (or non-functional siRNA (siR-NC) by western blot and qRT-PCR. qRT-PCR results were presented as the means ± SEM of three individual experiments in each group. *Non-specific band. **d** Western blot analysis of NF-κB2 (p100, p52) and RelB in subcellular fractions obtained from THP-1-derived macrophages. CE, cytoplasmic extract; NE, nuclear extract. *Non-specific band. **e** Phosphorylation of p100 in vivo. About 48 h postinfection, THP1 derived macrophages were incubated with a proteasome inhibitor, MG132 (30 mM), for 6 h and then lysed in RIPA buffer. The phosphorylation and total levels of p100 were monitored. **f** US31 promotes the in vivo ubiquitination of NF-κB2. THP1-derived macrophages at 48 h post infection with Ad-US31 (or Ad-GFP) and MG132 by IP and western blot

To identify the molecular mechanism of US31 during inflammation, we screened potential US31-interacting proteins using a customized protein binding array. 143 unique US31-interacting candidates were found in this screening assay (Table S12 and S13 Figure S3). Among these US31-interacting proteins, five proteins (NF-κB2, HSPA6, HSPA2, RNHI, and TXLNB) shown to be involved in immune system processes, and were chosen for further analysis. Notably, the functional groups assigned for this US31-interacting protein subset were largely related to NF-κB2 protein signaling (Fig. 4a). Co-immunoprecipitation (Co-IP) validated the direct interaction between US31 and NF-κB2. Endogenously expressed NF-κB2 protein was complexed with US31 following IP with an anti-US31 antibody (Fig. 4b). No detectable NF-κB2 was observed using control IgG. Furthermore, reciprocal Co-IP using an anti-NF-κB2 antibody confirmed this interaction. NF-κB2 is a member of the NF-κB transcription factor family that is well-known for its expression in multiple cell types and the role as a primary inflammation and immune function modulator^12–14^. To evaluate the effects of this US31 and NF-κB2 on immune function of mono-macrophages, we silenced NF-κB2 expression using si-RNA when US31 was expressed (Figure S4). The results showed that the NF-κB2 regulators and signaling molecules TNF-α, IL-8, CCL2, ICAM1, and RelB were significantly downregulated (Fig. 4c). These data directly indicated the change of cell function with US31expression was contributed the NF-kB2 activation in mono-macrophages.

### US31 contribute to the ubiquitination of NF-κB2 precursor and consequent activation

It is well know that, during the NF-κB2 activation, the p100 of NF-κB2 precursor is targeted for proteasomal processing to p52 of activated NF-κB2. Then the p52/RelB dimer then translocates into the nucleus to activate gene transcription. Subcellular fraction analysis by western blot indicates nuclear upregulation of both p52 of activated NF-κB2 and RelB protein (Fig. 4d). These data further confirmed the NF-κB activation by US31 protein. It has been demonstrated that the phosphorylation, polyubiquitination, and proteasomal processing of precursor p100 are required for NF-κB2 activation^15^. In vivo p100 phosphorylation (S866/870) and ubiquitination assays showed that p100 phosphorylation and ubiquitinated p100 accumulate in the presence of MG132 proteasome inhibitor when US31 is expressed (Fig. 4e, f). These data indicated that US31 promotes the ubiquitination of the phosphorylated p100 and consequently proteasomal processing to p52 and NF-κB activation. As the structural analysis of the US31 amino acid sequence suggests that it could be an E3 ubiquitin-protein ligase (ubr1), it was essential to evaluate the potential role of this protein in the processing of NF-κB2 during immune regulation. In addition, immunofluorescence microscopy of Ad-US31-infected THP-1-derived macrophages shows that US31 protein is primarily expressed in the cytoplasm and low amounts are present in the cell nucleus (Fig. 2d). As NF-κB2 protein has a similar expression pattern, with high levels in the cytoplasm and low levels in the cell nucleus^16,17^, it is possible that US31 could act as E3-like ligase and promote NF-κB2 activation.

## Discussion

Prior analysis of monocytes at 4 days after infection showed HCMV induces monocyte cell death, promotes inflammatory responses, induces cell cycle blockage, and participates in SLE development^5^. Moreover, transcriptome sequencing of monocytes after 4 h of infection indicated that HCMV also reprograms monocyte differentiation toward M1 macrophages^7^. However, the current understanding about HCMV gene expressions in PBMCs of SLE patients is limited.

Simultaneous performance of two library preparation methods to analyze global HCMV gene expression in the PBMCs of SLE patients afforded a more complete, unbiased understanding of viral gene expression in these samples. Specifically, poly(A)-Seq detected a total of 16 genes, whereas whole transcriptome sequencing detected 11 genes. Among these, 8 genes were detected in both sequencing methods.

Notably, the most of the detected viral genes in the PBMCs of SLE patients have been shown to be associated with HCMV latency^8^. For example, UL32, a major tegument protein that participates in virus assembly, maturation and release, gene expression and modulation, as well as host cell cycle modification and protein synthesis, is associated with latency-associated process^18^. Alternatively, UL36 participates in cell apoptosis, gene expression regulating, and encoding toxic proteins^19^. UL44^18,20^, UL50^21,22^, UL56^23^, UL82^24^, UL84^21^, UL95^21^, UL105^18^, and UL117^25^ also function in various latency-associated processes. Therefore, HCMV is in a latent infection state in the PBMCs of SLE patients.

There are currently three published studies concerning HCMV gene expression in monocyte or CD34+ haematopoietic cells.. Goodrum et al.^18^ used an in vitro model of HCMV latent infection in CD34+ cells, followed by HCMV cDNA array and found 68 HCMV genes distinct from HCMV productive infections. Similarly, Cheung et al.^26^ also used gene arrays to evaluate viral gene expression in latent HCMV-infected myeloid cells, and observed 37 differentially expressed genes. Lastly, high-throughput sequencing analysis of HCMV gene transcripts in experimental and natural HCMV CD14+ monocyte and CD34+ cell infection detected changes in a total of 20 genes^21^. Compared to these lists of differentially expressed genes (68, 37, and 20 genes, respectively), we detected only 7 (UL32, UL34, UL44, UL69, UL84, UL105, and UL123), 2 (UL56, UL123), and 6 (UL29, UL37, UL44, UL50, UL84, and UL95) of the same genes in our study, respectively (Table S14). The difference between the present and previously published findings may result from different detection methods, objects of study and patterns of experimental and natural HCMV infection. Furthermore, US31 expression, which our results indicate as a disease-related gene, was not investigated by the other studies.

However, the US31 gene identified during our analysis has not been investigated with regard to its expression mode and function. As expression of the proliferation-associated HCMV IE2 86 gene inhibits US31 expression, US31 may also be a HCMV latency-associated gene^27^. Notably, although neither Goodrum et al.^18^ or Cheung et al.^26^ detected the expression of US31, our data indicate that the positive rate of US31 gene expression in SLE patients is indeed higher compared to that in healthy control group, as confirmed using qPCR. Furthermore, compared with US31-negative SLE patients, the RBC numbers, complement C3 levels, and complement C4 levels in the US31-positive SLE patients were significantly lower, suggesting that US31 expression is involved in SLE development.

A study by Van Damme et al.^9^, the results of which we confirmed (Figure S5A), also shows high US31 expression in HCMV-infected monocyte-derived DCs, M1, and M2 cells. To explore the function of US31 in the mono-macrophage, recombinant adenovirus encoding US31 protein were used to infect THP-1 cells and THP-1-derived macrophages before transcriptome sequencing. Subsequent bioinformatic analysis showed that US31 mainly participates in the inflammatory response and a predominantly M1 macrophage phenotype. Using a HuProt™ human protein array combing with Co-IP experiments and reciprocal siRNA silencing test, we identified NF-κB2 is responsible for the immune function in mono-macrophages by direct interaction with US31.

**Fig. 5:**
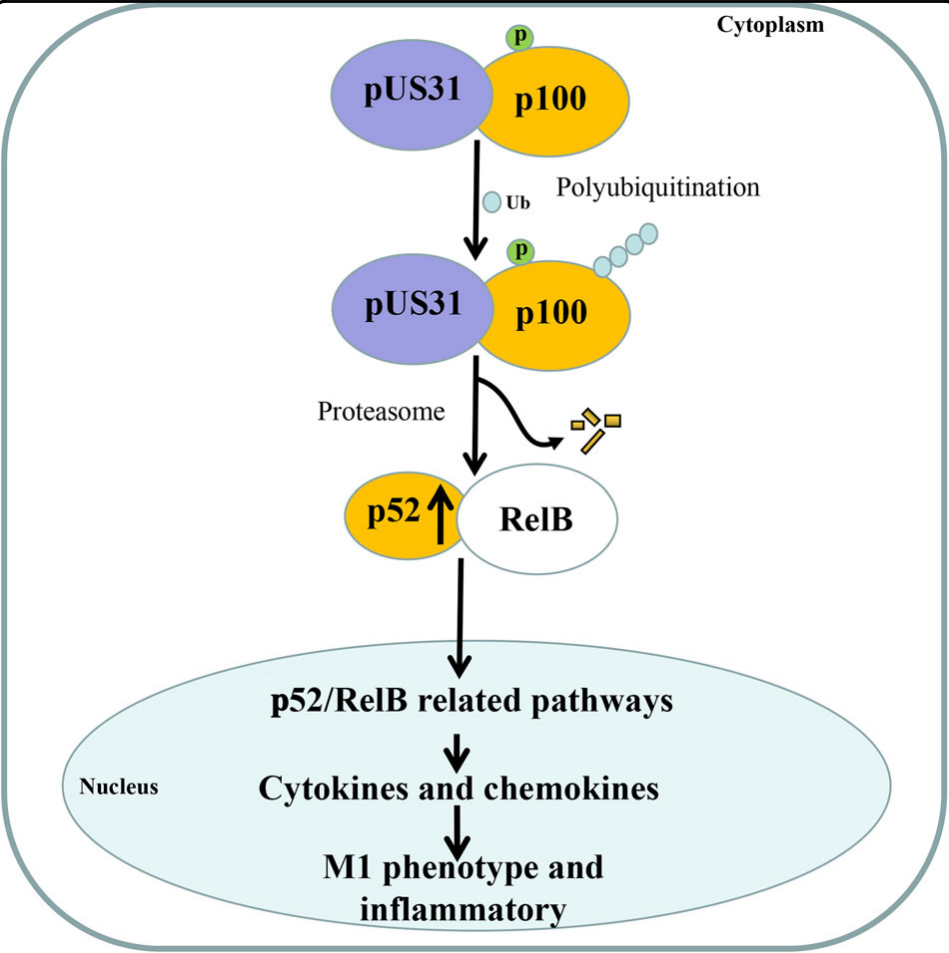
Schematic diagram of the functional consequences of HCMV-induced US31 expression in monocytes/macrophages Solid lines represent direct regulation

NF-κB regulates a number of key genes involved in cellular processes such as proliferation, apoptosis, and inflammation^28^. Traditionally, activation of NF-κB signaling is mediated through either canonical or non-canonical signaling pathways^15^. Non-canonical NF-κB signaling involves activation of the RelB/p52 NF-κB complex using a mechanism that relies on the polyubiquitination and proteasomal processing of the phosphorylated p100. The processing of p100 serves to generate p52 as well as induce the nuclear translocation of the RelB/p52 heterodimer^15^. Indeed, we found that US31 overexpression not only promoted the conversion of p100 to p52 as well as the translocation of the RelB/p52 complex into the nucleus, but also upregulated RelB and p100 expression (Figure S5B and S5C). Phosphorylated and ubiquitinated p100 accumulate in the presence of proteasome inhibitors MG132 when US31 is expressed. The inflammatory markers, including ICAM1, CCL2, IL-8, and TNF, downregulated after siRNA of NF-κB2 was transfected. These results clearly indicate that HCMV-induced US31 expression likely alters the expression of inflammation-related genes diseases via direct interaction with factors involved in the non-canonical NF-κB2 pathway. As the US31structure contained the homological domain of E3 ubiquitin-protein ligase (ubr1), it is possible that US31 could act as E3-like ligase and promote NF-κB2 activation. Additional details of this mechanism require further in-depth study (Fig. 5) as well as confirmation using infection with HCMV viruses with deficient/non-functional US31.

In summary, we provided the first global investigation of HCMV genes expression in PBMCs from SLE patients and demonstrated that US31 expression could change the immunological function of mono-macrophages and promote the M1 inflammatory response. In addition, we clarified for the first time that US31 could act as E3-like ligase and promote the ubiquitination of the phosphorylated p100 and proteasomal processing to consequently activate NF-κB2 pathway. The elucidation of this mechanism provides valuable insight for the future development of novel therapeutic options for SLE patients, with the ultimate goal of treating and/or preventing the development or HCMV-induced exacerbation of this prevalent autoimmune disease.

## Patients and methods

### Study population

A total of 87 SLE patients (73 women and 14 men), admitted to the Division of Rheumatology of the First and Second Affiliated Hospital of Wenzhou Medical University between October 2014 and May of 2016, were enrolled in this study. Written informed consent was obtained from the patients who participated in the study. The procedures of this study followed were in accordance with the ethical standards of the responsible committee on human experimentation (the hospital of the First and Second Affiliated Hospital of Wenzhou Medical University, China) and with the Helsinki Declaration of 1975, as revised in 2008. The Medical Ethical Committee of the hospital of the First and Second Affiliated Hospital of Wenzhou Medical University approved the consent procedure and subsequent testing protocols as well as anonymization and use of the data. All patients were in compliance with the classification standard set by the American College of Rheumatology in 1997^29^. The corresponding author, Xiangyang Xue, was responsible for anonymizing the data collected from participants and all of the data were used and analyzed in strictly anonymous form. Patients were aged from 21 to 77 years (mean 35.6 ± 13.9 years). All SLE patients were tested using a common clinical symptom survey and common testing indices and scored in accordance with the internationally accepted SLEDAI^30^. Blood samples from 79 healthy subjects (62 women and 17 men; 20–59-years-old) formed the normal population control group. Individuals infected with hepatitis B, hepatitis C, HIV, syphilis, Epstein-Barr virus, or who had other infections were excluded from both groups.

### Extraction of RNA from PBMCs

Blood (4 ml) was collected intravenously and placed in ethylenediamine tetra-acetic acid anticoagulant tubes. PBMCs were isolated by using human peripheral blood lymphocyte separation medium (Tianjin Hao Yang Biological Manufacture, Tianjin, China) according to the manufacturer’s protocol. Following isolation, PBMCs were washed twice with phosphate-buffered saline (PBS), and total RNA was extracted by using TRIzol reagent (Invitrogen Life Technologies®, Grand Island, NY, USA) according to the manufacturer’s protocol. Potential DNA contamination in the purified RNA samples was avoided by using DNAfree reagent (Ambion, Carlsbad, CA, USA) dissolved in diethyl pyrocarbonate-treated water. RNA samples were stored at −80 °C after spectrophotometer-based measurement of the concentration.

### Deep sequencing analysis of HCMV gene expression in PBMCs

The total RNA from the PBMCs of three SLE patients and three normal healthy controls (SLEDAI scores > 15, all women) was extracted to analyze HCMV gene expression. The anti-HCMV IgM and IgG levels in these three SLE patients were 0.296 ± 0.49 COI (mean ± standard error) and 269.867 ± 67.38 U/ml (mean ± standard error), respectively. Considering that the number of HCMV-infected PBMCs is low, and viral gene expression is low as well, we carried out mRNA (PolyA library) and whole transcriptome (strand-specific library) deep sequencing simultaneously in all six samples. The total RNA isolated from each sample was quantified and qualified using an Agilent 2100 Bioanalyzer (Agilent Technologies, Palo Alto, CA, USA), NanoDrop (Thermo Fisher Scientific Inc., Waltham, MA, USA), and 1% agarose gel electrophoresis. Total RNA (1 μg) with a RIN value above 7 was used for library preparation.

Poly(A) mRNA isolation was performed using an NEBNext Poly(A) mRNA Magnetic Isolation Module (NEB, Ipswich, MA, USA), whereas mRNA fragmentation and priming was conducted using NEBNext First Strand Synthesis Reaction Buffer and NEBNext Random Primers. First strand cDNA was synthesized using ProtoScript II Reverse Transcriptase, and second-strand cDNA was synthesized using Second Strand Synthesis Enzyme Mix. The purified double-stranded cDNA (performed using AxyPrep Mag PCR Clean-up [Axygen, Union City, CA, USA]) was then treated with End Prep Enzyme Mix to repair both ends and add a dA-tail in one reaction, followed by a T-A ligation to add adaptors to both ends. Size selection of Adaptor-ligated DNA was then performed using AxyPrep Mag PCR Clean-up (Axygen), and fragments of approximately 360 bp (with the approximate insert size of 300 bp) were recovered. Each sample was then amplified by PCR for 11 cycles using P5 and P7 primers, with both primers carrying sequences that would anneal to the flow cell for bridge PCR. The P7 primer also carried a six-base index that allows multiplexing. The PCR products were again cleaned up using AxyPrep Mag PCR Clean-up (Axygen), validated using an Agilent 2100 Bioanalyzer (Agilent Technologies), and quantified with a Qubit 2.0 Fluorometer (Invitrogen). Then, libraries with different indices were multiplexed and loaded onto an Illumina HiSeq instrument according to the manufacturer’s instructions (Illumina, San Diego, CA, USA). Sequencing was carried out using a 2 × 150 bp paired-end (PE) configuration. Image analysis and base calling were conducted with HiSeq Control Software (HCS)+OLB+GAPipeline-1.6 (Illumina).

In order to perform whole transcriptome deep sequencing, the rRNA was first removed from the total RNA using a Ribo-Zero™ rRNA Removal Kit (Human/Mouse/Rat) (Illumina). The ribosomal-depleted RNA was then fragmented and reverse-transcribed. First strand cDNA was synthesized using ProtoScript II Reverse Transcriptase with random primers and Actinomycin D. Second-strand cDNA was synthesized using Second Strand Synthesis Enzyme Mix (including dACG-TP/dUTP). Subsequent steps were the same those listed above for mRNA deep sequencing.

In order to detect HCMV sequences in the sequencing reads, Cutadapt software was first used for pre-processing of the raw data, allowing the low-quality data to be filtered and the contaminated and adapter sequences to be removed (settings other than default parameters: -q 20, 20; -m 75; --max-n = 0.1; -e 0.1). Further, Bowtie2 software was used to align the clean data reads with the viral genome from the publicly available reference genomes for Merlin (GenBank accession number: NC_006273.2) and TB40E (GenBank accession number: EF999921) (settings other than default parameters: --very-sensitive; --very-sensitive-local). Transcripts in fasta format were converted from known gff annotation file and indexed properly. Then, using the reference gene file, HTSeq (v0.6.1) was used to estimate gene expression levels from the pair-end clean data (no special parameters).

### PCR detection of HCMV genes in PBMCs

In order to quantitate 18 of the HCMV genes detected during the RNA-Seq analysis, we first obtained 17 HCMV strains from the National Center for Biotechnology Information (NCBI) database. The gene sequences corresponding to each strain are as follows: HAN31 (GenBank: JX512208.1), U11 (GenBank: GU179290.1), HAN20 (GenBank: GQ396663.1), HAN13 (GenBank: GQ221973.1), HAN38 (GenBank: GQ396662.1), Merlin (GenBank: AY446894.2), JP (GenBank: GQ221975.1), JHC (GenBank: HQ380895.1), AD169 (GenBank: FJ527563.1), 3301 (GenBank: GQ466044.1), 3157 (GenBank: GQ221974.1), VR1814 (GenBank: GU179289.1), AF1 (GenBank: GU179291.1), TR (GenBank: KF021605.1), U8 (GenBank: GU179288.1), Toledo (GenBank: GU937742.2), and Towne (GenBank: FJ616285.1). Homology analysis was carried out, and Primer 6.0 software was used to design upstream and downstream primers in the conserved regions of every gene. The designed primers were then aligned with the NCBI Primer-BLAST nr database to exclude the amplification of host genes and genomes of other herpesviruses. For quantitation of HCMV gene expression in PBMCs, reverse transcription was carried out on RNA samples from PBMCs isolated from four SLE patients. First-strand cDNA was reverse transcribed using a ReverTraAce® qPCR RT Kit (Toyobo®, Tokyo, Japan) from 1 µg of total RNA (denaturation at 65 °C for 5 min, incubation at 37 °C for 15 min, heat at 98 °C for 5 min). The reaction solution (X μl nuclease-free water, 2 μl 5 × RT buffer, 0.5 μl RT Enzyme Mix, 0.5 μl Primer Mix, and 1 μg RNA) was stored at −20 °C. The PCR reaction conditions consisted of denaturation at 95 °C for 5 min, followed by 35 cycles of 95 °C for 30 s, annealing temperature (gene specific) for 30 s, 72 °C for 30 s, and a final extension at 72 °C for 10 min. The PCR reaction solution consists of template (1 µg), primer 1 (10 µM), primer 2 (10 µM), Taq Polymerase (0.1 U/µl), and water up to 25 µl (TIANGEN Biotech C., Ltd., Beijing, China). The amplified products were electrophoresed on a 1.5% agarose gel containing ethidium bromide (EB) and the bands were photographed. Primers and reaction conditions are listed in Table S1.

### Quantitation of US31 in THP-1 cells infected with clinical viral strains

THP-1 cells (American Type Culture Collection (ATCC®), Manassas, VA, USA) were cultured using RPMI 1640 medium supplemented with 10% fetal bovine serum (FBS). HCMV clinical strains 3-2 were preserved by the Department of Microbiology and Immunology, Wenzhou Medical University. In order to detect US31 expression in HCMV-infected monocytes, THP-1 cells were infected with HCMV 3-2 (MOI = 5) for 3 h at 37 °C. The cells were cultured in fresh medium with 10% FBS continuously for 15 days. Cells were collected every day for RNA extraction and RT-PCR was carried out as described above to quantitate US31 expression.

### Testing of HCMV-specific serum IgG and IgM and anti-US31 serum level

HCMV-specific IgG and IgM antibodies in serum samples from SLE patients and normal controls were detected by enzyme-linked immunosorbent (ELISA) using an anti-CMV IgG and IgM test kit according to the instructions of the manufacturer (Roche, Mannheim, Germany). The ELISA tests were performed using a fully automated ELISA processor (Roche). Recombinant US31 purified from BL21/pET21a-US31 was used as antigen to detect the anti-US31 serum level in SLE patients and normal controls by ELISA.

### Preparation of recombinant US31 adenoviral overexpression vector

Upstream and downstream primers were designed according to the US31 gene sequence (GenBank accession number: GU937742). The upstream primer sequence was 5′-TCCAGACCCGGGACCGAATTCGCCACCATGTCGCTCTTGGAGCG-3′, and the downstream primer sequence was 5′-CGCTCGAGATCTGTAGTGTTGTTCACCCCGTGT-3′, with the underlined sequences containing an EcoRI restriction site. The PCR amplification conditions for the US31 gene were as follows: pre-denaturation at 95 °C for 5 min followed by 25 cycles of denaturation at 94 °C for 20 s, annealing at 55 °C for 20 s, and extension at 72 °C for 30 s, before a final extension of 72 °C for 10 min. The PCR product was verified using 1% agarose gel electrophoresis. The target segment was extracted with an agarose gel DNA extraction kit (TIANGEN) according to the manufacturer’s instructions. Ligation was carried out to obtain pHBAd-MCMV-GFP-US31 recombinant plasmids with 3 × flag tag, which were then transformed into DH5α competent cells and verified by restriction enzyme mapping and DNA sequencing (Huada, Shanghai, China).

HEK293T and COS-7 cells were obtained from the ATCC®. HEK293T cells were subcultured into 60-mm cell culture dishes. When the cells reached 70–80% confluency, Lipofiter^TM^ transfection reagent was used to transfect the recombinant pHBAd-MCMV-GFP-US31 vector plasmid into the cells. The cell culture medium was changed 6 h after transfection. Cells were observed for virus production every day, and when cytopathy was observed in the majority of the cells and these cells were detached from the plates, the viruses were harvested and stored at −80 °C. Then, HEK293T cells were subcultured onto 96-well plates at a density of 1 × 10^4^ cells/well. After the cells were adhered to the plate, the culture medium was removed and 100 μl of serially diluted viral supernatant (10^−6^–10^−13^) was added to each well. A total of 10 wells were subcultured with the same dilution as well as untreated negative controls. The cells were incubated at 37 °C, 5% CO_2_ for 10 days. The condition of the cells was observed on days 3 to 10. On day 10, the cytopathogenic effect (CPE) status of every well was observed under a microscope and compared with the negative controls. The number of positive wells for samples in each row was recorded. The recombinant adenovirus titer (d = log_10_, dilution = 1, s = sum of positivity ratios) was calculated based on the virus activity formula T = 10^1+d(s−0.5)^. Viral supernatants were sent to Hanbio Ltd. (Shanghai, China) for enrichment and purification. After purification, viral titers were 10^10^ PFU/ml and used for subsequent cell infection experiments. The Phyre2 Database (http://www.sbg.bio.ic.ac.uk/phyre2/html/page.cgi?id=index) was used for US31 amino acid structural analysis, whereas GalaxyWEB (http://galaxy.Seoklab.org/) was used to construct 3-dimensional models of US31.

### Induction of THP-1-derived macrophages and infection with US31 recombinant adenovirus

The induction protocol for THP-1 derived macrophages has been previously described^31^. Briefly, 3 × 10^5^ THP-1 cells/well were subcultured in 6-well plates, and PMA (300 ng/2 ml; Sigma-Aldrich, St. Louis, MO, USA) was added and incubated for 24 h to induce differentiation. When the proportion of cells adhering to the walls of the plate was 70–80%, PBS was used to wash the cells thrice to remove unsuccessfully induced cells floating in the culture medium. The remaining cells were THP-1-derived macrophages. Our previously reported multiplicity of infection (MOI) value for UL138 adenoviral transfection of monocytes was used as a reference^32^. In the present study, an MOI of 100, 500, or 1 for recombinant adenovirus ADU31 was used to infect THP-1 cells, THP-1-derived macrophages, or COS-7 and HEK293T cells, respectively. RT-qPCR and western blotting were used to quantitate US31 expression.

### US31 and inflammatory markers quantitation by quantitative real-time reverse transcription PCR (RT-qPCR)

In order to analyze US31 gene expression levels in PBMCs from patients with SLE and healthy controls, 1 µg of total PBMC-derived RNA was reverse transcribed with a ReverTraAce® qPCR RT Kit (Toyobo®, Tokyo, Japan). US31 expression was assessed by real-time PCR with iQ™ SYBR® Green Supermix (Bio-Rad, Berkeley, CA, USA) and specific primers (forward, 5′-GTATTCCTCGGGTTCCTC-3′; and reverse, 5′-TTTCCCCACAGTTAGACG-3′; 143 bp). Human *GAPDH* was used as an internal control (forward, 5′-AACTCTGGTAAAGTGGATATTG-3′; and reverse, 5′-GGTGGAATCATATTGGAACA-3′; 87 bp). The PCR program was 95 °C for 3 min, followed by 40 cycles of 95 °C for 10 s, 55 °C for 30 s, and 72 °C for 30 s. The RT-qPCR reaction solution contains 100 ng template, forward primer (10 µM), reverse primer (10 µM), 2 × iQ™ SYBR® Green Supermix, and nuclease-free water up to 10 µl. qPCR was performed using a CFX96 Touch™ Real-Time PCR Detection System (BioRad). Melting curves were generated for each real-time PCR to verify the specificity of each PCR reaction. Duplication was performed for accuracy judgment. The comparative viral copy (C_virus_) was normalized to the amount of *GAPDH* using the 2^−ΔCt^ method, with ΔCT = CT_UL31_– CT_GAPDH_. ICAM1, CCL2, IL-8, and TNF specific primers and program were previously reported^33–36^.

### Immunoblotting

Total protein was extracted from transfected HEK293T, COS-7, THP1, and macrophage cells using protein lysis buffer (Beyotime Institute of Biotechnology, Beijing, China) supplemented with protease inhibitor cocktail (Pierce, Rockford, IL, USA) at 4 °C for 20 min. Protein concentration was determined using a BCA assay kit. Protein samples (20 μg/lane) were separated using 10–12% sodium dodecyl sulfate (SDS)-polyacrylamide gel electrophoresis and then electrophoretically transferred to polyvinylidene difluoride membranes (Millipore, Billerica, MA, USA). After blocking with 5% skim milk for 1.5 h at 37 °C, the membranes were incubated with primary antibody at 4 °C overnight. Antibodies against NF-κB2 (p52/p100), phospho- NF-κB2 p100 (Ser 866/870), ubiquitin (P4D1), RelB (Cell Signaling Technology, Beverly, MA, USA), and GAPDH (GOOD HERE, Hangzhou, China) were diluted with Primary Antibody Dilution Buffer (Beyotime Institute of Biotechnology) at a 1:1000 dilution, whereas the US31 antibody was diluted 1:5000 (this antibody was a self-prepared rabbit anti-US31 antibody from our laboratory). The membranes were then washed with TBST buffer five times for 5 min each and incubated with secondary antibody (horseradish peroxidase (HRP)-conjugated goat anti-rabbit IgG (MULTI SCIENCES, Hangzhou, China)) for 1.5 h at 37 °C. The bands were detected using enhanced chemiluminescence and visualized with a Gel Doc 2000 (BioRad).

### Immunofluorescent localization of US31 protein

Co-localization of cellular proteins in the transfected cells was observed by immunofluorescence. Briefly, THP-1-derived macrophage (2 × 10^5^ cells/well in 6-well plates containing glass coverslips) were mock transfected or transfected with recombinant adenovirus encoding US31 for 48 h. Macrophage cells were then fixed in 4% paraformaldehyde at 37 °C for 10 min and permeabilized by 0.3% Triton X-100 at 37 °C for 10 min. After blocking the cells in PBS supplemented with 10% goat serum (Beyotime Institute of Biotechnology) for 60 min at 37 °C, the macrophages were stained with a rabbit antibody specific to US31. Secondary DyLight549–conjugated goat anti-rabbit antibody (MULTI SCIENCES) was applied in PBS supplemented with 10% goat serum for 1 h at 37 °C. Cells were subsequently incubated in Hoechst 33258 (Beyotime Institute of Biotechnology) for 5 min at room temperature. The coverslips were mounted on slides and visualized using a fluorescence microscope (Nikon C1–i, Tokyo, Japan).

### Effects of US31 expression on gene expression in THP-1 monocytes and THP-1-derived macrophages

Total RNA was extracted with TRIzol from THP-1 and THP-1-derived macrophages after transfection with adenovirus US31 or control adenovirus. The RNA was then purified with magnetic Agencourt Ampure beads (APN 000132, Beckman Coulter, Brea, CA, USA), and DNAfree reagent (Ambion, Carlsbad, CA, USA) was added to remove DNA contamination. Total RNA was quantified using a NanoDrop ND-1000, and RNA integrity was assessed by standard denaturing agarose gel electrophoresis. A poly(A) library preparation was used (after library preparation was completed, a Qubit® 2.0 Fluorometer was used for initial quantitation and to dilute the library to 1.5 ng/µl). Subsequently, an Agilent 2100 bioanalyzer was used to detect the insert size of the library, followed by qRT-PCR to quantitate the concentrations of the library and ensure quality (>2 nM). Paired-end sequencing was carried out based on the Illumina HiSeq/MiSeq technology platform. In order to eliminate biological variation, thresholds were set for the fold difference and significance level of the differentially expressed genes (|log2(FoldChange)| > 1 and *q-*value < 0.005). In addition, for genes showing statistical differences, the Annotation, Visualization and Integrated Discovery (DAVID) system was used to analyze the main biological processes and pathways. GO analysis was also used to carry out functional annotation, whereas KEGG was used for pathway analysis. In parallel with this analysis, software from Thomson Reuters Corporation (https://portal.geneGO.com/) was used to further analyze the differentially expressed genes using Pathway Maps, GO Processes, Process Networks, Diseases (by Biomarkers), and Network Statistics.

qPCR was performed to confirm the observed changes in expression in the RNA-Seq analysis. Total RNA from THP-1 and THP-1-derived macrophages transfected with adenovirus US31 or control adenovirus was extracted with TRIzol reagent. RT-PCR was performed using a PrimeScript^TM^ RT reagent kit (TaKaRa, Otsu, Japan) following the manufacturer’s instructions. A customized PCR array from CT Bioscience^37,38^ (Changzhou, China) was used to compare the expression profile of a selected group of genes in the adenoviral US31 (Ad-US31)-transfected vs. Ad-GFP-transfected THP-1 cells/THP-1-derived macrophages. Target mRNAs included molecules potentially involved in chemokine/cytokine signaling. PCR amplification of these cytokines/chemokines was performed for 10 min at 94 °C, followed by 45 cycles of 94 °C for 15 s, annealing at 60 °C for 15 s, and extension at 72 °C for 20 s in a Roche light cycler 480 II PCR machine (Roche, Basel, Switzerland), using the SYBR Premix Ex Taq kit (TaKaRa). β2-microglobulin (*B2M*), actin, beta (*ACTB*), ribosomal protein L27 (*RPL27*), hypoxanthine phosphoribosyltransferase 1 (*HPRT1*), and ornithine decarboxylase antizyme 1 (*OAZ1*) were used as housekeeping genes for normalization. The relative quantitation method used was based on the Ct value of the test samples, and 2^−△△Ct^ was used to express the relative expression of each cytokine/chemokine/receptor, whereby △△Ct = (Ct_cytokine/chemokine_−Ct_reference gene_) _experimental group_−(Ct_cytokine/chemokine_−Ct_reference gene_) _control group_. If the fold change (2^−△△Ct^) was less than 1, then then inverse (−1/2^−△△Ct^) was used.

### Protein array screening of US31-binding proteins

The Johns Hopkins Medical Institutions Protein Microarray Core produced the protein binding microarray chips with 19394 individual human GST-and His6-tagged full-length proteins. Recombinant US31 purified from BL21/pET21a-US31 (1.5 μg/μl) was labeled using an Alexa-Flour-647 microscale protein labeling kit (A30009, Molecular Probes/Invitrogen). The data were extracted from the microarray images with GenePix ProTM 6.0. Background was defined as signals less than 20% of the maximum signal and removed from the subsequent analysis. The signal-to-noise ratios (SNR = F635 median/B635 median) were first calculated for all of the spots to generate the candidate list of US31-binding proteins. The SNR of a protein was calculated as the average of two duplicated spots.

### Bioinformatics analysis of the US31 interactome

The PANTHER (Protein Analysis through Evolutionary Relationships) (http://www.pantherdb.org/) system, a unique resource that classifies genes and proteins according to their functions, was used to classify the US31 interactome. To evaluate protein overexpression, GO term enrichment, KEGG pathways, and Pfam domain families, DAVID 6.7 (https://david.ncifcrf.gov/) was used. The default human proteome was used as the background list. The *P*-value (EASE-score, Fisher-Pvalue, or Benjamini-Hochberg false discovery rate correction) indicates the significance of the pathway correlated with the conditions. The distribution of cellular components, molecular functions, and biological processes of the US31 interactome were also analyzed. The lower the *P*-value, the more significant the GO term (*P*-value < 0.05 was considered statistically significant).

### Immunoprecipitation

THP-1-derived macrophage cells were infected with the recombinant Ad-US31 or Ad-GFP control in 6-well dishes for 3 h. The media was then replaced, and the cells were incubated as indicated. At 48 h post-transfection, the cells were lysed for 5 min on ice with immunoprecipitation (IP) buffer (Pierce) supplemented with cocktail protease inhibitors (Pierce). The lysates were then centrifuged to clear cell debris at 13,000×*g* for 10 min at 4 °C. Then, 10 μg of rabbit antibodies specific to US31, mouse antibodies specific to Flag (Sigma), or anti-NF-κB2 was combined with disuccinimidyl suberate binding to protein A/G plus agarose. Then, a Pierce® Crosslink Immunoprecipitation Kit was used to collect the immune complexes as described in the manufacturer’s protocol. The eluted protein complexes were then resuspended in reducing SDS sample buffer, heated at 95 °C for 10 min, and analyzed by immunoblotting.

### Ubiquitination assays

For in vivo ubiquitination analysis, 30 μM MG132 (Sigma- Aldrich) was applied to the cells. Ad-US31-infected- THP1 derived macrophages were treated with MG132 starting at 36 hpi, and harvested at 42 hpi. DMSO was applied as the solvent control. Cell lysates were subjected to NF-κB2-directed IP (Cell Signaling Technology) followed by IB using a polyclonal rabbit antibody for ubiquitin (Cell Signaling Technology).

### Statistical analysis

Data are expressed as the means ± standard errors and statistical significance was tested using a t-test and ANOVA between the groups. Numerical data are represented as *n* (%) and statistical significance was tested using the chi-square test or Fisher’s exact probability. A *P < *0.05 was considered statistically significant. All statistical results of the data were analyzed with SPSS 16.0 (Chicago, IL, USA).

## Electronic supplementary material

SUPPLEMENTAL MATERIAL

SUPPLEMENTAL Figure S1

SUPPLEMENTAL Figure S2

SUPPLEMENTAL Figure S3

SUPPLEMENTAL Figure S4

SUPPLEMENTAL Figure S5
