# Supplementary material for: The cytomegalovirus protein US31 induces inflammation through mono-macrophages in systemic lupus erythematosus by promoting NF-κB2 activation
Source: Cell Death Dis. 2018 Jan 24;9(2):104. doi: 10.1038/s41419-017-0122-4 (PMC5833803; doi:10.1038/s41419-017-0122-4)
Supplement: Supplementary file 1 — SUPPLEMENTAL MATERIAL [file 41419_2017_122_MOESM1_ESM.doc]

**Supplementary Information**

The cytomegalovirus protein US31 induces inflammation through mono-macrophages in systemic lupus erythematosus by promoting NF-κB2 activation

**Supplementary Figure Legends**

**Figure S1 (A)** Cluster analysis of HCMV transcripts detected along with a bar graph representing the HCMV gene expression for each positive sample using poly(A) library preparation (mRNA sequencing). (**B**) PCR detection of US31 after 5 days of HCMV infection in THP-1 cells. NC: negative controls (No HCMV infection). (**C**, **D**) Detection of IgG and IgM antihuman cytomegalovirus in sera from SLE and control patients. Sera were detected by enzyme-linked immunosorbent assay (ELISA) using anti-CMV IgG and IgM test kit.****P* < 0.001; NS, no statistical significance. The results are expressed by 50% quantile (25% quantile, 75% quantile). (**E**) Anti-US31 detection between SLE patients and the healthy control group. The results are expressed by 50% quantile (25% quantile, 75% quantile). ****P* < 0.001. ROC curve analysis to calculate the discriminative power of US31 antibody titer to predict positive rates of SLE patients.

**Figure S2** PCR validation. (**A**, **B**) We performed qRT-PCR on a subset of 18 randomly chosen genes of THP-1-derived macrophages (15 upregulated and 3 downregulated). These genes include 10 cytokines and their receptors (**A**) as well as 8 chemokines and their receptors (**B**). (**C**) Pearson correlation was used to verify the RNA-Seq data. X-axis: −ΔΔCT values from qPCR comparing Ad-US31-infected THP-1-derived macrophages or Ad-GFP-infected macrophages (control); Y-axis: log2 (fold change) between infected or mock-infected THP-1-derived macrophages via RNA-Seq. Pearson correlation coefficient (R) based on all genes is shown. (**D**) qRT-PCR analysis showing a subset of 7 randomly chosen genes expressed in infected THP-1 cells using the specified method. (**E**) Pearson correlation was used to verify the RNA-Seq data. X-axis: −ΔΔCT values from qPCR comparing Ad-US31-infected THP-1 cells or Ad-GFP-infected cells; Y-axis: log2 (fold change) between infected or mock-infected THP-1 cells via RNA-Seq.

**Figure S3** PANTHER classification of the US31-interacting proteins divided into biological processes (**A**), molecular function (**B**), cellular component (**C**), and signaling pathways (**D**).

**Figure S4** The protein expression level of NF-κB2 was decreased when transfected with siRNA against NF-κB2 in THP1 derived macrophages. GAPDH served as a loading control. *****Non specific band (NS).

**Figure S5** (**A**) Expression of US31 in monocyte-derived M1, M2, and DC cells after HCMV infection. The height of the profile indicates the number of sequenced reads, shown on the y-axis. A, B, C, D, E and F represent different samples. (**B**) qPCR verification of NF-κB2 and RelB upregulation at 48 h after US31 infection in THP-1-derived macrophages. **P* < 0.05. NS: not statistically significance. NF-κB2 and RelB upregulation is shown in fold increase compared to non-infected cells (set equal to 1). (**C**) Western blot analysis of the US31-associated inflammation-related proteins NF-κB2 (p100, p52) and RelB in THP-1-derived macrophages 48 h post-infection with Ad-US31 or Ad-GFP (indicated as US31+/−). GAPDH was used as a loading control. *Non-specific band.

**Supplementary Tables**

**Table S1** Primers and reaction conditions for amplifying 18 viral ORFs

| Gene | Sequences | | annealing temperature（s） | Cycles | Product(bp) |
| --- | --- | --- | --- | --- | --- |
|  | Forward Prime（5’-3’） | Reverse Number（5’-3’） |  |  |  |
| UL29^*^ | ATGTCCGGCCGTCGCAAGG | TCACCTACGCTTTTTGAACGGC | 62 | 35 | 1080 |
| UL32 | ATTACGACGAAGAGGAAAAGCG | TCGGCACTGGAGCCAAAGA | 64 | 35 | 187 |
| UL34 | CGACGAGTTGACCAAACG | GACCGATCTGGAAGAGCAT | 60 | 35 | 290 |
| UL36 | CTGTTCGCAAGGTAAGCC | GCGTTCCAGTAGTCGTCATA | 58 | 35 | 370 |
| UL37^*^ | TTCTTCATCCTCCCTTTCCTC | GCGGGTTTCACTTCTTTCTTT | 57 | 35 | 337 |
| UL44 | CTCTTCGCTGGCTAATGC | TGATCTTGTGCTGCTCGTAT | 60 | 35 | 236 |
| UL50 | CTCATCGTCACGCTCAAGG | CATCCGCATCGTCCCATA | 62 | 35 | 467 |
| UL56 | CACGAGGATACGGCTTTG | GCCAGACTGACGCTGAAA | 57 | 35 | 161 |
| UL82 | CGACAGCAGTTTGGAGGCA | AGGTGCGGGTACGGATTGT | 62 | 35 | 273 |
| UL84 | AACGGCGAGTCTCAAACC | GAGTGATAAGCGCACGATG | 63 | 35 | 279 |
| UL95 | AAACGCCCGACGAGAATT | ACAAAGCGACGGCAAAGG | 63 | 35 | 287 |
| UL105 | CTCGGGTTCCTCTTTGTGCT | GCGTGGTGCGTTTGTCTTT | 60 | 35 | 337 |
| UL112^*^ | ATGGATCTCCCTACTACCGTCG | TTAATCGTCGAAAAACGCCGCG | 62 | 35 | 2055 |
| UL117 | CACAGAGGCTGAGGGGAAAC | ACTGGAGGAGCCCGTTGAA | 58 | 35 | 135 |
| UL123 | TAATACAAGCCATCCACA | TAGATAAGGTTCATGAGCCT | 55 | 35 | 117 |
| US3^*^ | ATGAAGCCGGTGTTGGTGC | TTAAATAAATCGCAGACGGGCGC | 62 | 35 | 561 |
| US31 | AAGCCACGTTGAGATTCAG | CTAGTGCTACTTTCCCCACA | 64 | 35 | 324 |
| TRS1^*^ | CTCCCAAGAACCCAGAAAGTC | TCACCCTAGCCACCAAACA | 58 | 35 | 349 |

*From Cheung AK. et al.^1^

| **Table S2 Correlation between intracellular HCMV infection in PBMC and clinical indicators in SLE patients.**   | Clinical indicators^#^ | Normal range | US31 | | | | --- | --- | --- | --- | --- | | ＋ | － | P Value* | | (n=52) | (n=23) | | Age (year) | ／ | 36.41±14.93 | 34.00±11.73 | 0.496 | | Female | ／ | 45(86.54%) | 21(91.30%) | 0.841 | | SLEDAI scores | ≤4 score | 8.50 (4.00,16.00) | 8..00(5.00,15.00) | 0.940 | | First onset | ／ | 33(64.71%) | 18(78.26%) | 0.244 | | Anti-ds-DNA antibody | Negative | 23(57.5%) | 8(42.5%) | 0.781 | | Anti-Rib-P antibody | Negative | 20(54.1%) | 10(66.7%) | 0.404 | | Anti-Simth antibody | Negative | 9(24.3%) | 5(33.3%) | 0.750 | | Anti-U1RNP antibody | Negative | 16(43.2%) | 8(53.3%) | 0.508 | | Anti-histone antibody | Negative | 4(10.8%) | 2(13.3%) | 1.000 | | Anti-SS-A antibody | Negative | 23(62.2%) | 11(73.3%) | 0.443 | | Anti-SS-B antibody | Negative | 8(21.6%) | 6(40.0%) | 0.313 | | Direct Coomb's test | Negative | 12(42.9%) | 5(41.7%) | 0.944 | | Indirect Coomb's test | Negative | 6(21.4%) | 1(8.3%) | 0.586 | | β2-GP1 | 0-20 RU/mL | 3.08 (1.08，12.9) | 2.70(1.38，6.63) | 0.790 | | WBC | 3.50～9.50 ×10^9^/L | 5.20(2.83，6.98) | 5.33(3.48，9.29) | 0.550 | | RBC | 3.80～5.10 ×10^12^/L | 3.51±0.71 | 3.88±0.68 | **0.043** | | Neutrophils | 1.80～6.30 ×10^9^/L | 3.14(1.71，4.58) | 3.44(2.03，5.61) | 0.534 | | Lymphocytes | 1.10～3.20 ×10^9^/L | 1.05(0.73，1.91) | 1.23(0.76，1.41) | 0.941 | | Monocytes | 0.10～0.60 ×10^9^/L | 0.41(0.32，0.71) | 0.48(0.35，0.64) | 0.818 | | Platelets | 125～350×10^9^/L | 196.98±78.99 | 169.14±74.83 | 0.167 | | Hemoglobin | 115～150 g/L | 110.78±20.08 | 100.45±19.05 | 0.046 | | ALT | 7～40 U/L | 26.85±30.30 | 18.41±10.99 | 0.210 | | AST | 13～35 U/L | 37.41±58.24 | 22.82±11.17 | 0.250 | | Alb | 40.0～55.0 g/L | 31.38±7.79 | 31.31±8.57 | 0.972 | | Total IgG | 7.00～16.00 g/L | 16.60±10.24 | 14.13±4.19 | 0.184 | | Total IgM | 0.40～2.30 g/L | 1.18±0.80 | 1.17±0.84 | 0.972 | | Total IgA | 0.70～4.00 g/L | 2.80±1.74 | 2.65±1.19 | 0.729 | | Complement C3 | 0.79～1.52 g/L | 0.47±0.20 | 0.61±0.28 | **0.028** | | Complement C4 | 0.16～0.38 g/L | 0.07±0.05 | 0.12±0.07 | **0.022** | | Proteinuria | Negative | 17(35.4%) | 11(47.8%) | 0.317 | | Hematuria | Negative | 28(58.3%) | 12(52.2%) | 0.624 | | 24-h urine protein | <0.15 g/24h | 0.92(0.31,4.96) | 0.70(0.13,3.60) | 0.499 | | CRP | 0.00～8.00 mg/L | 14.70±18.47 | 8.07±7.33 | 0.065 | | ESR | 0～20 mm/h | 29.52±26.52 | 19.11±15.67 | 0.117 | | Creatinine | 35～80 umol/L | 52.50(47.00,68.75) | 51.00(40.00,85.75) | 0.569 | | BUN | 2.8～7.2 mmol/L | 4.60(3.53,7.00) | 4.45(3.58,8.00) | 0.950 | | Calcium | 2.10～2.60 mmol/L | 2.03±0.18 | 2.05±0.22 | 0.844 | | D-dimer | 0.00～0.50 mg/L | 1.40(0.90,2.61) | 1.41(0.98,2.24) | 0.983 | | LAC | 0.75～1.25 mg/L | 1.07±0.33 | 0.97±0.32 | 0.320 |   ^#^ESR erythrocyte sedimentation rate, CRP C-reactive protein, RBC red blood cell, WBC white blood cell, Alb albumin, ALT alanine aminotransferase, AST aspartate transaminase, BUN blood urea nitrogen, β2-GP1 β2-glycoprotein 1.  *Significant differences are indicated in bold. *P*<0.05 was considered significant. The correlation between intracellular HCMV infection in PBMCs and clinical indicators in SLE patients was compared using the chi-square test and Fisher’s or Student's t test or non-parametric Mann-Whitney U test for exact probability. The clinical indicators did not present a normal distribution and the results were expressed by 50% quantile (25% quantile, 75% quantile), or the results were expressed by the means ± SD. Numerical data are represented as n (%). |
| --- | --- | --- | --- | --- | --- | --- | --- | --- | --- | --- | --- | --- | --- | --- | --- | --- | --- | --- | --- | --- | --- | --- | --- | --- | --- | --- | --- | --- | --- | --- | --- | --- | --- | --- | --- | --- | --- | --- | --- | --- | --- | --- | --- | --- | --- | --- | --- | --- | --- | --- | --- | --- | --- | --- | --- | --- | --- | --- | --- | --- | --- | --- | --- | --- | --- | --- | --- | --- | --- | --- | --- | --- | --- | --- | --- | --- | --- | --- | --- | --- | --- | --- | --- | --- | --- | --- | --- | --- | --- | --- | --- | --- | --- | --- | --- | --- | --- | --- | --- | --- | --- | --- | --- | --- | --- | --- | --- | --- | --- | --- | --- | --- | --- | --- | --- | --- | --- | --- | --- | --- | --- | --- | --- | --- | --- | --- | --- | --- | --- | --- | --- | --- | --- | --- | --- | --- | --- | --- | --- | --- | --- | --- | --- | --- | --- | --- | --- | --- | --- | --- | --- | --- | --- | --- | --- | --- | --- | --- | --- | --- | --- | --- | --- | --- | --- | --- | --- | --- | --- | --- | --- | --- | --- | --- | --- | --- | --- | --- | --- | --- | --- | --- | --- | --- | --- | --- | --- | --- | --- | --- | --- | --- | --- | --- | --- | --- | --- | --- | --- | --- | --- | --- | --- | --- | --- |
|

**Table S3** The total of 133 differentially expressed genes*

| Gene Name | Description | Read Count | | log2FoldChange | p value | padj (q value) |
| --- | --- | --- | --- | --- | --- | --- |
|  |  | AD-US31-THP1 | AD-GFP-THP1 |  |  |  |
| CCL2 | chemokine (C-C motif) ligand 2 | 1286.70 | 95.69 | 3.75 | 2.65E-30 | 6.54E-26 |
| CXCL10 | chemokine (C-X-C motif) ligand 10 | 1128.37 | 90.95 | 3.63 | 4.30E-28 | 5.31E-24 |
| MMP9 | matrix metallopeptidase 9 (gelatinase B, 92kDa gelatinase, 92kDa type IV collagenase) | 909.87 | 182.85 | 2.32 | 8.32E-14 | 6.85E-10 |
| RSAD2 | radical S-adenosyl methionine domain containing 2 | 1859.86 | 457.59 | 2.02 | 1.25E-12 | 7.70E-09 |
| IL4I1 | interleukin 4 induced 1 | 3923.43 | 992.86 | 1.98 | 1.73E-12 | 8.54E-09 |
| IFIT2 | interferon-induced protein with tetratricopeptide repeats 2 | 3579.32 | 944.54 | 1.92 | 6.64E-12 | 2.73E-08 |
| HMOX1 | heme oxygenase (decycling) 1 | 1063.98 | 263.37 | 2.01 | 1.51E-11 | 5.32E-08 |
| ISG20 | interferon stimulated exonuclease gene 20kDa | 369.44 | 66.32 | 2.48 | 2.54E-11 | 7.85E-08 |
| CCL8 | chemokine (C-C motif) ligand 8 | 174.16 | 17.05 | 3.35 | 3.57E-11 | 9.78E-08 |
| GBP1 | guanylate binding protein 1, interferon-inducible | 274.44 | 42.63 | 2.69 | 5.50E-11 | 1.36E-07 |
| IFIT3 | interferon-induced protein with tetratricopeptide repeats 3 | 5149.96 | 1460.87 | 1.82 | 1.27E-10 | 2.78E-07 |
| ADAMDEC1 | ADAM-like, decysin 1 | 262.83 | 41.68 | 2.66 | 1.35E-10 | 2.78E-07 |
| CXCL13 | chemokine (C-X-C motif) ligand 13 | 112.94 | 6.63 | 4.09 | 1.55E-10 | 2.94E-07 |
| IL8 | interleukin 8 | 748.38 | 188.53 | 1.99 | 1.84E-10 | 3.24E-07 |
| IL1B | interleukin 1, beta | 411.66 | 87.16 | 2.24 | 2.86E-10 | 4.71E-07 |
| SLAMF8 | SLAM family member 8 | 787.43 | 204.64 | 1.94 | 3.08E-10 | 4.75E-07 |
| CYBB | cytochrome b-245, beta polypeptide | 3851.65 | 1148.23 | 1.75 | 3.80E-10 | 5.52E-07 |
| IFI27 | interferon, alpha-inducible protein 27 | 1632.91 | 480.32 | 1.77 | 4.90E-10 | 6.48E-07 |
| LACC1 | laccase (multicopper oxidoreductase) domain containing 1 | 858.15 | 231.16 | 1.89 | 4.98E-10 | 6.48E-07 |
| MAFB | v-maf musculoaponeurotic fibrosarcoma oncogene homolog B (avian) | 1227.59 | 357.16 | 1.78 | 8.58E-10 | 1.06E-06 |
| C1orf204 | chromosome 1 open reading frame 204 | 299.77 | 61.58 | 2.28 | 3.00E-09 | 3.53E-06 |
| MIR320A | microRNA 320a | 104.50 | 434.85 | -2.06 | 3.39E-09 | 3.80E-06 |
| ENSG00000204044.5 | ENSG00000204044.5 | 248.05 | 46.42 | 2.42 | 4.08E-09 | 4.38E-06 |
| RGL1 | ral guanine nucleotide dissociation stimulator-like 1 | 309.27 | 66.32 | 2.22 | 4.88E-09 | 5.02E-06 |
| SLAMF7 | SLAM family member 7 | 271.27 | 54.95 | 2.30 | 6.18E-09 | 6.10E-06 |
| IFI44 | interferon-induced protein 44 | 2306.35 | 779.70 | 1.56 | 1.65E-08 | 1.56E-05 |
| ICAM1 | intercellular adhesion molecule 1 | 868.71 | 270.95 | 1.68 | 2.10E-08 | 1.92E-05 |
| IFI44L | interferon-induced protein 44-like | 6584.44 | 2187.51 | 1.59 | 2.81E-08 | 2.48E-05 |
| DDX58 | DEAD (Asp-Glu-Ala-Asp) box polypeptide 58 | 1857.74 | 638.54 | 1.54 | 3.35E-08 | 2.85E-05 |
| IFIT1 | interferon-induced protein with tetratricopeptide repeats 1 | 4249.59 | 1470.34 | 1.53 | 3.72E-08 | 3.06E-05 |
| TBC1D17 | TBC1 domain family, member 17 | 392.66 | 104.21 | 1.91 | 4.36E-08 | 3.47E-05 |
| EBI3 | Epstein-Barr virus induced 3 | 222.72 | 46.42 | 2.26 | 6.28E-08 | 4.84E-05 |
| CXCL11 | chemokine (C-X-C motif) ligand 11 | 234.33 | 52.11 | 2.17 | 1.07E-07 | 7.90E-05 |
| RND3 | Rho family GTPase 3 | 263.88 | 62.53 | 2.08 | 1.09E-07 | 7.90E-05 |
| IGFBP3 | insulin-like growth factor binding protein 3 | 395.83 | 112.74 | 1.81 | 1.65E-07 | 0.000117 |
| GBP4 | guanylate binding protein 4 | 900.37 | 308.85 | 1.54 | 1.89E-07 | 0.00013 |
| PDGFRL | platelet-derived growth factor receptor-like | 124.55 | 18.95 | 2.72 | 2.74E-07 | 0.000183 |
| CLEC7A | C-type lectin domain family 7, member A | 175.22 | 36.00 | 2.28 | 4.24E-07 | 0.000276 |
| HERC6 | HECT and RLD domain containing E3 ubiquitin protein ligase family member 6 | 778.99 | 272.85 | 1.51 | 5.07E-07 | 0.000321 |
| TRIM22 | tripartite motif containing 22 | 2782.39 | 1074.34 | 1.37 | 5.87E-07 | 0.000363 |
| OASL | 2'-5'-oligoadenylate synthetase-like | 1262.42 | 471.80 | 1.42 | 6.05E-07 | 0.000364 |
| IFIH1 | interferon induced with helicase C domain 1 | 3314.38 | 1296.02 | 1.35 | 8.49E-07 | 0.000499 |
| MS4A3 | membrane-spanning 4-domains, subfamily A, member 3 (hematopoietic cell-specific) | 366.27 | 989.07 | -1.43 | 9.24E-07 | 0.00053 |
| LAMP3 | lysosomal-associated membrane protein 3 | 99.22 | 14.21 | 2.80 | 1.11E-06 | 0.00062 |
| TNFAIP3 | tumor necrosis factor, alpha-induced protein 3 | 1564.31 | 617.70 | 1.34 | 1.59E-06 | 0.000875 |
| FGL2 | fibrinogen-like 2 | 402.16 | 129.79 | 1.63 | 1.64E-06 | 0.000878 |
| STAT2 | signal transducer and activator of transcription 2, 113kDa | 2677.90 | 1077.18 | 1.31 | 1.69E-06 | 0.000886 |
| BATF2 | basic leucine zipper transcription factor, ATF-like 2 | 356.77 | 111.79 | 1.67 | 1.74E-06 | 0.000887 |
| MX2 | myxovirus (influenza virus) resistance 2 (mouse) | 4505.03 | 1794.35 | 1.33 | 1.76E-06 | 0.000887 |
| ETV7 | ets variant 7 | 185.77 | 44.53 | 2.06 | 1.98E-06 | 0.000977 |
| ZFP36L1 | ZFP36 ring finger protein-like 1 | 281.83 | 82.42 | 1.77 | 2.09E-06 | 0.001011 |
| EPSTI1 | epithelial stromal interaction 1 (breast) | 1811.30 | 738.01 | 1.30 | 2.87E-06 | 0.001363 |
| GBP5 | guanylate binding protein 5 | 356.77 | 115.58 | 1.63 | 3.14E-06 | 0.001461 |
| USP18 | ubiquitin specific peptidase 18 | 2676.84 | 1107.49 | 1.27 | 3.41E-06 | 0.001557 |
| DMXL2 | Dmx-like 2 | 2836.23 | 1177.60 | 1.27 | 3.72E-06 | 0.001669 |
| IL1RN | interleukin 1 receptor antagonist | 1496.75 | 612.01 | 1.29 | 3.92E-06 | 0.001728 |
| IL15RA | interleukin 15 receptor, alpha | 215.33 | 58.74 | 1.87 | 4.09E-06 | 0.001773 |
| MT2A | metallothionein 2A | 910.93 | 360.01 | 1.34 | 4.74E-06 | 0.002019 |
| DTX4 | deltex homolog 4 (Drosophila) | 848.65 | 333.48 | 1.35 | 5.02E-06 | 0.0021 |
| LILRB2 | leukocyte immunoglobulin-like receptor, subfamily B (with TM and ITIM domains), member 2 | 347.27 | 116.53 | 1.58 | 6.56E-06 | 0.002701 |
| MPEG1 | macrophage expressed 1 | 1370.09 | 573.17 | 1.26 | 7.56E-06 | 0.003062 |
| SAT1 | spermidine/spermine N1-acetyltransferase 1 | 293.44 | 94.74 | 1.63 | 8.08E-06 | 0.003217 |
| HERC5 | HECT and RLD domain containing E3 ubiquitin protein ligase 5 | 1128.37 | 468.96 | 1.27 | 9.03E-06 | 0.003541 |
| IL10RA | interleukin 10 receptor, alpha | 1171.65 | 488.85 | 1.26 | 9.20E-06 | 0.00355 |
| MIR122 | microRNA 122 | 155.16 | 430.11 | -1.47 | 1.02E-05 | 0.003803 |
| MIR3591 | microRNA 3591 | 155.16 | 430.11 | -1.47 | 1.02E-05 | 0.003803 |
| BIRC3 | baculoviral IAP repeat containing 3 | 195.27 | 54.95 | 1.83 | 1.16E-05 | 0.004262 |
| IFI16 | interferon, gamma-inducible protein 16 | 3197.22 | 1390.76 | 1.20 | 1.17E-05 | 0.004262 |
| C19orf66 | chromosome 19 open reading frame 66 | 1059.76 | 444.32 | 1.25 | 1.23E-05 | 0.004415 |
| APOL3 | apolipoprotein L, 3 | 131.94 | 30.32 | 2.12 | 1.30E-05 | 0.004603 |
| CCL3 | chemokine (C-C motif) ligand 3 | 116.11 | 24.63 | 2.24 | 1.34E-05 | 0.004652 |
| LILRA6 | leukocyte immunoglobulin-like receptor, subfamily A (with TM domain), member 6 | 64.39 | 8.53 | 2.92 | 1.48E-05 | 0.00508 |
| HELZ2 | helicase with zinc finger 2, transcriptional coactivator | 6206.55 | 2660.26 | 1.22 | 1.66E-05 | 0.005602 |
| CHST2 | carbohydrate (N-acetylglucosamine-6-O) sulfotransferase 2 | 557.32 | 219.79 | 1.34 | 1.83E-05 | 0.006104 |
| TMEM106A | transmembrane protein 106A | 370.49 | 135.48 | 1.45 | 2.13E-05 | 0.007001 |
| NRP1 | neuropilin 1 | 1093.54 | 471.80 | 1.21 | 2.15E-05 | 0.007001 |
| PLA2G7 | phospholipase A2, group VII (platelet-activating factor acetylhydrolase, plasma) | 165.72 | 45.47 | 1.87 | 2.29E-05 | 0.007339 |
| ENSG00000267607.1 | ENSG00000267607.1 | 201.61 | 61.58 | 1.71 | 2.81E-05 | 0.008893 |
| NCF1 | neutrophil cytosolic factor 1 | 296.61 | 104.21 | 1.51 | 2.88E-05 | 0.008993 |
| LILRB1 | leukocyte immunoglobulin-like receptor, subfamily B (with TM and ITIM domains), member 1 | 231.16 | 74.84 | 1.63 | 2.95E-05 | 0.009103 |
| LPXN | leupaxin | 795.87 | 339.16 | 1.23 | 3.17E-05 | 0.009665 |
| OAS1 | 2'-5'-oligoadenylate synthetase 1, 40/46kDa | 2526.95 | 1152.02 | 1.13 | 3.36E-05 | 0.010123 |
| UBE2L6 | ubiquitin-conjugating enzyme E2L 6 | 2639.90 | 1212.65 | 1.12 | 3.97E-05 | 0.011695 |
| NFE2L3 | nuclear factor (erythroid-derived 2)-like 3 | 664.99 | 280.43 | 1.25 | 3.98E-05 | 0.011695 |
| SAMSN1 | SAM domain, SH3 domain and nuclear localization signals 1 | 254.38 | 87.16 | 1.55 | 4.06E-05 | 0.011792 |
| IFIT5 | interferon-induced protein with tetratricopeptide repeats 5 | 671.32 | 285.16 | 1.24 | 4.46E-05 | 0.012816 |
| LGALS3BP | lectin, galactoside-binding, soluble, 3 binding protein | 1337.36 | 606.33 | 1.14 | 4.58E-05 | 0.013004 |
| PTAFR | platelet-activating factor receptor | 515.10 | 211.27 | 1.29 | 4.79E-05 | 0.013443 |
| ENSG00000248323.1 | ENSG00000248323.1 | 44.33 | 4.74 | 3.23 | 5.24E-05 | 0.014548 |
| IFITM1 | interferon induced transmembrane protein 1 | 3794.65 | 1757.40 | 1.11 | 5.36E-05 | 0.0147 |
| PAX5 | paired box 5 | 379.99 | 148.74 | 1.35 | 6.02E-05 | 0.016333 |
| NFKBIA | nuclear factor of kappa light polypeptide gene enhancer in B-cells inhibitor, alpha | 2177.57 | 1021.28 | 1.09 | 6.48E-05 | 0.0174 |
| ZC3H12A | zinc finger CCCH-type containing 12A | 605.88 | 259.58 | 1.22 | 6.78E-05 | 0.018011 |
| TDO2 | tryptophan 2,3-dioxygenase | 123.50 | 32.21 | 1.94 | 7.11E-05 | 0.018672 |
| NFKB2 | nuclear factor of kappa light polypeptide gene enhancer in B-cells 2 (p49/p100) | 1044.98 | 476.53 | 1.13 | 7.32E-05 | 0.018875 |
| SAMD9 | sterile alpha motif domain containing 9 | 4324.53 | 2022.67 | 1.10 | 7.34E-05 | 0.018875 |
| IL7R | interleukin 7 receptor | 230.11 | 79.58 | 1.53 | 7.43E-05 | 0.018888 |
| CMPK2 | cytidine monophosphate (UMP-CMP) kinase 2, mitochondrial | 4525.08 | 2114.56 | 1.10 | 7.50E-05 | 0.018888 |
| APOL6 | apolipoprotein L, 6 | 3974.09 | 1875.82 | 1.08 | 8.37E-05 | 0.02074 |
| JUP | junction plakoglobin | 274.44 | 101.37 | 1.44 | 8.51E-05 | 0.02074 |
| HRH1 | histamine receptor H1 | 449.66 | 185.69 | 1.28 | 8.62E-05 | 0.02074 |
| DUSP6 | dual specificity phosphatase 6 | 970.04 | 443.38 | 1.13 | 8.65E-05 | 0.02074 |
| GPR84 | G protein-coupled receptor 84 | 317.72 | 122.21 | 1.38 | 8.66E-05 | 0.02074 |
| GMPR | guanosine monophosphate reductase | 117.16 | 30.32 | 1.95 | 8.74E-05 | 0.02074 |
| EMR1 | egf-like module containing, mucin-like, hormone receptor-like 1 | 79.17 | 16.11 | 2.30 | 8.83E-05 | 0.020767 |
| HESX1 | HESX homeobox 1 | 186.83 | 60.63 | 1.62 | 8.98E-05 | 0.020923 |
| CD274 | CD274 molecule | 67.55 | 12.32 | 2.46 | 9.29E-05 | 0.021442 |
| DHX58 | DEXH (Asp-Glu-X-His) box polypeptide 58 | 599.54 | 262.43 | 1.19 | 0.0001 | 0.023485 |
| C3AR1 | complement component 3a receptor 1 | 384.22 | 156.32 | 1.30 | 0.00011 | 0.02461 |
| IFI30 | interferon, gamma-inducible protein 30 | 1610.75 | 769.28 | 1.07 | 0.00011 | 0.025334 |
| ANGPTL6 | angiopoietin-like 6 | 279.72 | 106.11 | 1.40 | 0.00012 | 0.025646 |
| FGD2 | FYVE, RhoGEF and PH domain containing 2 | 1328.92 | 630.01 | 1.08 | 0.00012 | 0.025646 |
| ARHGAP31 | Rho GTPase activating protein 31 | 683.99 | 306.95 | 1.16 | 0.00012 | 0.026108 |
| SOD2 | superoxide dismutase 2, mitochondrial | 1972.80 | 953.07 | 1.05 | 0.00013 | 0.027369 |
| ENSG00000245532.4 | ENSG00000245532.4 | 1825.02 | 882.02 | 1.05 | 0.00013 | 0.028239 |
| SLCO2B1 | solute carrier organic anion transporter family, member 2B1 | 104.50 | 26.53 | 1.98 | 0.00013 | 0.028239 |
| EPHB2 | EPH receptor B2 | 108.72 | 28.42 | 1.94 | 0.00014 | 0.029919 |
| NFE2 | nuclear factor (erythroid-derived 2), 45kDa | 48.55 | 156.32 | -1.69 | 0.00014 | 0.03027 |
| ATF5 | activating transcription factor 5 | 1985.46 | 968.23 | 1.04 | 0.00015 | 0.031751 |
| SIGLEC1 | sialic acid binding Ig-like lectin 1, sialoadhesin | 6021.84 | 2872.47 | 1.07 | 0.00016 | 0.032947 |
| OAS3 | 2'-5'-oligoadenylate synthetase 3, 100kDa | 21269.06 | 8667.63 | 1.30 | 0.00017 | 0.034703 |
| TMCC3 | transmembrane and coiled-coil domain family 3 | 69.67 | 14.21 | 2.29 | 0.00017 | 0.035042 |
| AQP1 | aquaporin 1 (Colton blood group) | 2.11 | 30.32 | -3.84 | 0.00017 | 0.035042 |
| IFI6 | interferon, alpha-inducible protein 6 | 9849.21 | 4536.08 | 1.12 | 0.0002 | 0.039297 |
| ENSG00000225963.2 | ENSG00000225963.2 | 146.72 | 46.42 | 1.66 | 0.00022 | 0.04256 |
| NCOA7 | nuclear receptor coactivator 7 | 1941.13 | 962.54 | 1.01 | 0.00022 | 0.04256 |
| CSRNP1 | cysteine-serine-rich nuclear protein 1 | 516.16 | 231.16 | 1.16 | 0.00022 | 0.042742 |
| ACSL1 | acyl-CoA synthetase long-chain family member 1 | 2483.68 | 1237.29 | 1.01 | 0.00022 | 0.043345 |
| PLSCR1 | phospholipid scramblase 1 | 1888.35 | 939.81 | 1.01 | 0.00024 | 0.045302 |
| HAVCR2 | hepatitis A virus cellular receptor 2 | 139.33 | 43.58 | 1.68 | 0.00024 | 0.046191 |
| FTH1 | ferritin, heavy polypeptide 1 | 22953.70 | 9421.75 | 1.28 | 0.00025 | 0.046645 |
| NFKBIZ | nuclear factor of kappa light polypeptide gene enhancer in B-cells inhibitor, zeta | 299.77 | 122.21 | 1.29 | 0.00025 | 0.047013 |
| AIM2 | absent in melanoma 2 | 38.00 | 4.74 | 3.00 | 0.00025 | 0.047013 |

*Fold change > 2 and q<0.05

**Table S4 The total of 92 differentially expressed genes***

| Gene Name | Description | readcount_AD_US31 | readcount_AD_GFP | log2.Fold_change. | pvalue | qvalue |
| --- | --- | --- | --- | --- | --- | --- |
| ONECUT2 | one cut homeobox 2 | 9.04 | 36.10 | -1.9981 | 1.61E-05 | 0.0020717 |
| USP2 | ubiquitin specific peptidase 2 | 141.78 | 70.49 | 1.0082 | 4.49E-06 | 0.00066373 |
| SP100 | SP100 nuclear antigen | 100.37 | 49.83 | 1.0102 | 0.00011024 | 0.011316 |
| APOC1 | apolipoprotein C-I | 95.00 | 47.15 | 1.0107 | 0.00016737 | 0.015991 |
| IFI35 | interferon-induced protein 35 | 104.85 | 51.95 | 1.013 | 7.43E-05 | 0.0080585 |
| OCSTAMP | osteoclast stimulatory transmembrane protein | 211.21 | 102.46 | 1.0436 | 7.67E-09 | 1.94E-06 |
| LRRC28 | leucine rich repeat containing 28 | 121.46 | 58.88 | 1.0448 | 1.16E-05 | 0.0015662 |
| GPC4 | glypican 4 | 600.90 | 287.76 | 1.0623 | 4.22E-23 | 4.37E-20 |
| PIK3AP1 | phosphoinositide-3-kinase adaptor protein 1 | 101.75 | 48.68 | 1.0635 | 4.57E-05 | 0.005283 |
| HELZ2 | helicase with zinc finger 2, transcriptional coactivator | 402.64 | 191.60 | 1.0714 | 3.25E-16 | 1.83E-13 |
| PML | promyelocytic leukemia | 97.94 | 46.29 | 1.0811 | 4.94E-05 | 0.0056468 |
| ERGIC1 | endoplasmic reticulum-golgi intermediate compartment (ERGIC) 1 | 84.67 | 39.84 | 1.0876 | 0.00014858 | 0.01437 |
| TRAF1 | TNF receptor-associated factor 1 | 74.83 | 35.18 | 1.0886 | 0.00035858 | 0.03061 |
| RGS2 | regulator of G-protein signaling 2, 24kDa | 169.13 | 79.48 | 1.0895 | 7.89E-08 | 1.61E-05 |
| MMP10 | matrix metallopeptidase 10 (stromelysin 2) | 605.00 | 283.45 | 1.0938 | 2.13E-24 | 2.52E-21 |
| STAT2 | signal transducer and activator of transcription 2, 113kDa | 142.23 | 66.39 | 1.0992 | 6.95E-07 | 0.00011906 |
| FGR | Gardner-Rasheed feline sarcoma viral (v-fgr) oncogene homolog | 247.97 | 115.46 | 1.1028 | 4.96E-11 | 1.76E-08 |
| DUSP1 | dual specificity phosphatase 1 | 138.51 | 64.30 | 1.1071 | 8.29E-07 | 0.00013912 |
| ATF3 | activating transcription factor 3 | 167.67 | 77.35 | 1.1161 | 4.74E-08 | 1.02E-05 |
| PARP10 | poly (ADP-ribose) polymerase family, member 10 | 185.96 | 85.34 | 1.1237 | 7.23E-09 | 1.85E-06 |
| DTX3L | deltex 3-like (Drosophila) | 176.73 | 80.04 | 1.1429 | 1.04E-08 | 2.56E-06 |
| IL1B | interleukin 1, beta | 7176.45 | 3235.46 | 1.1493 | 1.78E-295 | 2.21E-291 |
| GIPC3 | GIPC PDZ domain containing family, member 3 | 98.37 | 44.14 | 1.1561 | 1.61E-05 | 0.0020717 |
| CCL20 | chemokine (C-C motif) ligand 20 | 518.17 | 231.12 | 1.1648 | 2.17E-23 | 2.35E-20 |
| RGS16 | regulator of G-protein signaling 16 | 194.32 | 86.14 | 1.1738 | 8.14E-10 | 2.50E-07 |
| NR4A3 | nuclear receptor subfamily 4, group A, member 3 | 62.39 | 27.61 | 1.1761 | 0.0004902 | 0.038781 |
| LY6E | lymphocyte antigen 6 complex, locus E | 311.77 | 137.85 | 1.1773 | 6.16E-15 | 3.06E-12 |
| NES | nestin | 185.80 | 82.13 | 1.1778 | 1.71E-09 | 4.98E-07 |
| KLHL6 | kelch-like family member 6 | 93.36 | 41.23 | 1.1792 | 1.92E-05 | 0.0024192 |
| TDO2 | tryptophan 2,3-dioxygenase | 595.62 | 261.59 | 1.1871 | 1.79E-27 | 2.79E-24 |
| PHLDA3 | pleckstrin homology-like domain, family A, member 3 | 73.37 | 32.21 | 1.1877 | 0.00013751 | 0.0136 |
| KIAA1199 | KIAA1199 | 62.20 | 27.02 | 1.2028 | 0.00038721 | 0.032386 |
| CSPG4 | chondroitin sulfate proteoglycan 4 | 66.92 | 28.94 | 1.2095 | 0.00021693 | 0.020183 |
| TRIM14 | tripartite motif containing 14 | 55.30 | 23.25 | 1.2499 | 0.00054622 | 0.042402 |
| SAMD9L | sterile alpha motif domain containing 9-like | 141.72 | 59.41 | 1.2543 | 2.84E-08 | 6.48E-06 |
| SLC15A3 | solute carrier family 15, member 3 | 121.36 | 50.04 | 1.2782 | 1.80E-07 | 3.47E-05 |
| RGS1 | regulator of G-protein signaling 1 | 496.05 | 202.98 | 1.2892 | 2.21E-26 | 3.04E-23 |
| EIF2AK2 | eukaryotic translation initiation factor 2-alpha kinase 2 | 215.50 | 87.31 | 1.3034 | 1.56E-12 | 6.27E-10 |
| TRIM69 | tripartite motif containing 69 | 52.57 | 21.28 | 1.3051 | 0.00047403 | 0.037854 |
| CD83 | CD83 molecule | 150.17 | 60.73 | 1.306 | 3.41E-09 | 9.33E-07 |
| COL22A1 | collagen, type XXII, alpha 1 | 160.06 | 64.18 | 1.3184 | 7.73E-10 | 2.40E-07 |
| SLC11A1 | solute carrier family 11 (proton-coupled divalent metal ion transporters), member 1 | 141.45 | 56.11 | 1.3341 | 5.27E-09 | 1.36E-06 |
| LPL | lipoprotein lipase | 843.62 | 332.67 | 1.3425 | 1.31E-46 | 3.61E-43 |
| IL4I1 | interleukin 4 induced 1 | 76.53 | 29.70 | 1.3653 | 1.20E-05 | 0.0016087 |
| RASSF4 | Ras association (RalGDS/AF-6) domain family member 4 | 57.99 | 22.43 | 1.3708 | 0.000132 | 0.013222 |
| STAC2 | SH3 and cysteine rich domain 2 | 73.56 | 27.70 | 1.409 | 1.07E-05 | 0.0014516 |
| SAMD9 | sterile alpha motif domain containing 9 | 146.23 | 54.87 | 1.4141 | 4.79E-10 | 1.53E-07 |
| BCL2A1 | BCL2-related protein A1 | 108.65 | 40.55 | 1.422 | 7.02E-08 | 1.44E-05 |
| CMPK2 | cytidine monophosphate (UMP-CMP) kinase 2, mitochondrial | 42.97 | 15.74 | 1.4493 | 0.00057709 | 0.044109 |
| IGFBP3 | insulin-like growth factor binding protein 3 | 143.42 | 52.48 | 1.4503 | 3.12E-10 | 1.02E-07 |
| PARP14 | poly (ADP-ribose) polymerase family, member 14 | 194.13 | 70.78 | 1.4556 | 2.10E-13 | 8.69E-11 |
| IFIH1 | interferon induced with helicase C domain 1 | 93.55 | 32.41 | 1.529 | 1.16E-07 | 2.27E-05 |
| PLSCR1 | phospholipid scramblase 1 | 121.55 | 42.08 | 1.5303 | 1.50E-09 | 4.43E-07 |
| HERC6 | HECT and RLD domain containing E3 ubiquitin protein ligase family member 6 | 62.82 | 21.63 | 1.5383 | 1.28E-05 | 0.0017017 |
| GM2A | GM2 ganglioside activator | 196.80 | 67.54 | 1.5429 | 9.84E-15 | 4.70E-12 |
| MYADM | myeloid-associated differentiation marker | 37.79 | 12.67 | 1.5765 | 0.00056184 | 0.043209 |
| IL1RN | interleukin 1 receptor antagonist | 729.22 | 243.55 | 1.5821 | 3.68E-52 | 1.31E-48 |
| TFPI2 | tissue factor pathway inhibitor 2 | 346.83 | 115.16 | 1.5906 | 6.76E-26 | 8.84E-23 |
| LIF | leukemia inhibitory factor | 114.42 | 37.84 | 1.5965 | 1.35E-09 | 4.09E-07 |
| EPSTI1 | epithelial stromal interaction 1 (breast) | 70.59 | 22.96 | 1.6206 | 1.47E-06 | 0.00023951 |
| STAT1 | signal transducer and activator of transcription 1, 91kDa | 263.92 | 83.78 | 1.6554 | 2.96E-21 | 2.45E-18 |
| CHIT1 | chitinase 1 (chitotriosidase) | 42.57 | 13.47 | 1.6603 | 0.00013952 | 0.01368 |
| IFITM3 | interferon induced transmembrane protein 3 | 98.97 | 31.06 | 1.6719 | 5.25E-09 | 1.36E-06 |
| LIPA | lipase A, lysosomal acid, cholesterol esterase | 268.80 | 82.98 | 1.6956 | 2.34E-22 | 2.15E-19 |
| HSPB7 | heat shock 27kDa protein family, member 7 (cardiovascular) | 87.32 | 26.85 | 1.7016 | 2.74E-08 | 6.35E-06 |
| MSR1 | macrophage scavenger receptor 1 | 174.68 | 53.34 | 1.7115 | 2.89E-15 | 1.53E-12 |
| XAF1 | XIAP associated factor 1 | 88.64 | 26.52 | 1.7408 | 1.23E-08 | 2.94E-06 |
| CCL3L3 | chemokine (C-C motif) ligand 3-like 3 | 552.11 | 162.78 | 1.762 | 1.16E-46 | 3.60E-43 |
| IRF7 | interferon regulatory factor 7 | 68.49 | 20.16 | 1.7646 | 4.26E-07 | 7.62E-05 |
| CCL4 | chemokine (C-C motif) ligand 4 | 60.23 | 17.71 | 1.766 | 2.08E-06 | 0.00033409 |
| NR1H3 | nuclear receptor subfamily 1, group H, member 3 | 42.81 | 12.05 | 1.8286 | 4.07E-05 | 0.0047731 |
| SIGLEC1 | sialic acid binding Ig-like lectin 1, sialoadhesin | 145.20 | 37.66 | 1.9469 | 2.72E-15 | 1.47E-12 |
| CCL3L1 | chemokine (C-C motif) ligand 3-like 1 | 152.89 | 39.52 | 1.952 | 4.51E-16 | 2.49E-13 |
| OAS3 | 2'-5'-oligoadenylate synthetase 3, 100kDa | 539.32 | 132.05 | 2.0301 | 2.06E-55 | 1.02E-51 |
| PARP9 | poly (ADP-ribose) polymerase family, member 9 | 133.39 | 32.24 | 2.0488 | 4.28E-15 | 2.22E-12 |
| CSF1 | colony stimulating factor 1 (macrophage) | 5196.55 | 1222.69 | 2.0875 | 0 | 0 |
| ISG15 | ISG15 ubiquitin-like modifier | 111.16 | 25.99 | 2.0966 | 3.45E-13 | 1.41E-10 |
| OAS1 | 2'-5'-oligoadenylate synthetase 1, 40/46kDa | 76.50 | 15.82 | 2.2733 | 1.96E-10 | 6.52E-08 |
| OAS2 | 2'-5'-oligoadenylate synthetase 2, 69/71kDa | 120.71 | 23.43 | 2.3653 | 2.46E-16 | 1.42E-13 |
| DDX58 | DEAD (Asp-Glu-Ala-Asp) box polypeptide 58 | 73.21 | 13.20 | 2.4713 | 5.48E-11 | 1.92E-08 |
| HERC5 | HECT and RLD domain containing E3 ubiquitin protein ligase 5 | 25.36 | 4.57 | 2.4728 | 0.0001132 | 0.011525 |
| IFIT5 | interferon-induced protein with tetratricopeptide repeats 5 | 26.87 | 4.83 | 2.4749 | 7.02E-05 | 0.0076785 |
| IFI44 | interferon-induced protein 44 | 31.42 | 5.45 | 2.5272 | 1.34E-05 | 0.0017663 |
| IFIT3 | interferon-induced protein with tetratricopeptide repeats 3 | 131.09 | 21.87 | 2.5839 | 2.08E-19 | 1.52E-16 |
| OASL | 2'-5'-oligoadenylate synthetase-like | 25.63 | 4.27 | 2.5843 | 6.79E-05 | 0.0075002 |
| RSAD2 | radical S-adenosyl methionine domain containing 2 | 61.64 | 8.69 | 2.8258 | 8.34E-11 | 2.88E-08 |
| IFIT2 | interferon-induced protein with tetratricopeptide repeats 2 | 30.40 | 3.83 | 2.9883 | 2.65E-06 | 0.00041729 |
| MX1 | myxovirus (influenza virus) resistance 1, interferon-inducible protein p78 (mouse) | 29.27 | 3.54 | 3.049 | 3.25E-06 | 0.00049857 |
| MX2 | myxovirus (influenza virus) resistance 2 (mouse) | 70.35 | 7.60 | 3.2099 | 1.39E-13 | 5.97E-11 |
| USP18 | ubiquitin specific peptidase 18 | 37.39 | 3.95 | 3.243 | 6.03E-08 | 1.25E-05 |
| IFIT1 | interferon-induced protein with tetratricopeptide repeats 1 | 97.40 | 8.16 | 3.5769 | 7.32E-20 | 5.51E-17 |
| TRIM22 | tripartite motif containing 22 | 13.41 | 1.03 | 3.7002 | 0.00060479 | 0.046084 |

*Fold change > 2 and q < 0.05

**Table S5 Transcriptome sequencing and differential gene expression analysis in Ad-US31 and Ad-GFP-transfected THP1 cells**

| Category | Term | Number of genes | Fold Enrichment | P Value |
| --- | --- | --- | --- | --- |
| GO | GO:0051607~defense response to virus | 23 | 20.532 | 1.07E-22 |
|  | GO:0009615~response to virus | 17 | 22.764 | 2.36E-17 |
|  | GO:0006954~inflammatory response | 23 | 8.939 | 7.40E-15 |
|  | GO:0060337~type I interferon signaling pathway | 13 | 29.92 | 1.17E-14 |
|  | GO:0045071~negative regulation of viral genome replication | 10 | 36.825 | 4.70E-12 |
|  | GO:0006955~immune response | 19 | 6.648 | 4.05E-10 |
|  | GO:0060333~interferon-gamma-mediated signaling pathway | 9 | 18.672 | 2.45E-08 |
|  | GO:0071222~cellular response to lipopolysaccharide | 9 | 11.732 | 9.32E-07 |
|  | GO:0032480~negative regulation of type I interferon production | 6 | 29.46 | 1.57E-06 |
|  | GO:0045087~innate immune response | 13 | 4.453 | 3.26E-05 |
|  | GO:0031663~lipopolysaccharide-mediated signaling pathway | 5 | 23.015 | 6.04E-05 |
|  | GO:0070098~chemokine-mediated signaling pathway | 6 | 12.448 | 1.15E-04 |
|  | GO:0006935~chemotaxis | 7 | 8.452 | 1.74E-04 |
|  | GO:0002230~positive regulation of defense response to virus by host | 4 | 26.781 | 4.16E-04 |
|  | GO:0071356~cellular response to tumor necrosis factor | 6 | 8.034 | 8.81E-04 |
|  | GO:0060326~cell chemotaxis | 5 | 11.331 | 9.60E-04 |
| Kegg | hsa05164:Influenza A | 11 | 6.826 | 3.19E-06 |
|  | hsa04380:Osteoclast differentiation | 9 | 7.418 | 2.17E-05 |
|  | hsa04668:TNF signaling pathway | 8 | 8.149 | 4.47E-05 |
|  | hsa04064:NF-kappa B signaling pathway | 7 | 8.687 | 1.28E-04 |
|  | hsa05162:Measles | 8 | 6.494 | 1.88E-04 |
|  | hsa04060:Cytokine-cytokine receptor interaction | 10 | 4.694 | 2.12E-04 |
|  | hsa05168:Herpes simplex infection | 9 | 5.31 | 2.31E-04 |
|  | hsa04062:Chemokine signaling pathway | 9 | 5.224 | 2.59E-04 |

**Table S6 Transcriptome sequencing and differential gene expression analysis in Ad-US31 and Ad-GFP-infected THP1-derived macrophages**

| Category | Term | Number of genes | Fold Enrichment | P Value |
| --- | --- | --- | --- | --- |
| GO | GO:0060337~type I interferon signaling pathway | 18 | 54.916 | 1.94E-25 |
|  | GO:0009615~response to virus | 19 | 33.726 | 9.54E-23 |
|  | GO:0051607~defense response to virus | 20 | 23.667 | 5.88E-21 |
|  | GO:0045071~negative regulation of viral genome replication | 11 | 53.695 | 4.84E-15 |
|  | GO:0060333~interferon-gamma-mediated signaling pathway | 9 | 24.751 | 2.53E-09 |
|  | GO:0006955~immune response | 13 | 6.029 | 1.33E-06 |
|  | GO:0035456~response to interferon-beta | 4 | 86.78 | 1.03E-05 |
|  | GO:0045087~innate immune response | 12 | 5.449 | 1.06E-05 |
|  | GO:0006954~inflammatory response | 9 | 5.152 | 1.25E-04 |
|  | GO:0030593~neutrophil chemotaxis | 5 | 14.792 | 3.47E-04 |
|  | GO:0032480~negative regulation of type I interferon production | 4 | 26.034 | 4.60E-04 |
|  | GO:0034340~response to type I interferon | 3 | 83.681 | 5.23E-04 |
|  | GO:0035457~cellular response to interferon-alpha | 3 | 65.085 | 8.91E-04 |
|  | GO:0035455~response to interferon-alpha | 3 | 58.577 | 0.001 |
|  | GO:0002548~monocyte chemotaxis | 4 | 18.596 | 0.001 |
|  | GO:0032727~positive regulation of interferon-alpha production | 3 | 48.814 | 0.001 |
| Kegg | hsa05168:Herpes simplex infection | 14 | 11.747 | 5.22E-11 |
|  | hsa05164:Influenza A | 13 | 11.473 | 4.66E-10 |
|  | hsa05162:Measles | 11 | 12.7 | 7.07E-09 |
|  | hsa05160:Hepatitis C | 10 | 11.546 | 1.11E-07 |
|  | hsa04620:Toll-like receptor signaling pathway | 6 | 8.692 | 5.22E-04 |
|  | hsa04062:Chemokine signaling pathway | 7 | 5.779 | 0.001 |
|  | hsa04064:NF-kappa B signaling pathway | 5 | 8.825 | 0.002 |
|  | hsa05323:Rheumatoid arthritis | 5 | 8.725 | 0.002 |
|  | hsa04060:Cytokine-cytokine receptor interaction | 7 | 4.673 | 0.003 |

**Table S7 NFKB activation affects hallmarks of inflammatory diseases**

|  | Gene Name | readcount_ADUS31 | readcount_ADGFP | Fold Change | pvalue |
| --- | --- | --- | --- | --- | --- |
|  | ICAM1 | 868.71 | 270.95 | 3.21 | 0.000 |
|  | IL1β | 411.66 | 87.16 | 4.72 | 0.000 |
|  | CCL2 | 1286.70 | 95.69 | 13.45 | 0.000 |
| THP1 | IL-8 | 748.38 | 188.53 | 3.97 | 0.000 |
|  | TNF | 62.28 | 59.69 | 1.04 | 0.939 |
|  | RelB | 694.5430172 | 341.0587885 | 2.04 | 0.001 |
|  | ICAM1 | 116.99 | 72.64 | 1.61 | 0.004 |
|  | IL1β | 7176.45 | 3235.46 | 2.22 | 0.000 |
| THP1 derived macrophage | CCL2 | 39.44 | 16.00 | 2.46 | 0.003 |
|  | IL-8 | 10322.93 | 10228.90 | 1.01 | 0.003 |
|  | TNF | 47.15 | 31.47 | 1.50 | 0.120 |
|  | RelB | 81.40803185 | 61.82419755 | 1.32 | 0.180 |

**Table S8 Comparison of M1 genes expressed in ADUS31-infected THP1 cells#**

| Category and Full Gene Name | Gene Title | Fold Change |
| --- | --- | --- |
| Membrane receptors | | |
| chemokine (C-C motif) receptor 7 | CCR7 | 2.8 |
| interleukin 2 receptor, alpha | IL2RA | nc |
| interleukin 15 receptor, alpha | IL15RA | 3.7 |
| interleukin 7 receptor | IL7R | 2.9 |
| Cytokines and chemokines | | |
| chemokine (C-X-C motif) ligand 11 | CXCL11 | 4.5 |
| chemokine (C-C motif) ligand 19 | CCL19 | nc |
| chemokine (C-X-C motif) ligand 10 | CXCL10 | 12.4 |
| chemokine (C-X-C motif) ligand 9 | CXCL9 | 3.9 |
| tumor necrosis factor | TNF | nc |
| chemokine (C-C motif) ligand 5 | CCL5 | 1.7 |
| chemokine (C-C motif) ligand 15 | CCL15 | nc |
| interleukin 12B | IL12B | nc |
| interleukin 15 | IL15 | 1.7 |
| interleukin 6 (interferon, beta 2) | IL6 | nc |
| chemokine (C-C motif) ligand 20 | CCL20 | nc |
| Apoptosis-related genes | | |
| BCL2-related protein A1 | BCL2A1 | 2.5 |
| Fas cell surface death receptor | FAS | nc |
| baculoviral IAP repeat containing 3 | BIRC3 | 3.6 |
| growth arrest and DNA-damage-inducible, gamma | GADD45G | 3.3 |
| Solute carriers | | |
| solute carrier family 7, member 5 | SLC7A5 | nc |
| solute carrier family 2, member 6 | SLC2A6 | 2 |
| solute carrier family 31, member 2 | SLC31A2 | 1.8 |
| Enzymes | | |
| phospholipase A1 member A | PLA1A | nc |
| 2'-5'-oligoadenylate synthetase-like | OASL | 2.7 |
| chitinase 3-like 2 | CHI3L2 | -1.8 |
| hydroxysteroid (11-beta) dehydrogenase 1 | HSD11B1 | nc |
| adenylate kinase 3 | AK3 | nc |
| sphingosine kinase 1 | SPHK1 | 1.9 |
| 6-phosphofructo-2-kinase/fructose-2,6-biphosphatase 3 | PFKFB3 | nc |
| proteasome (prosome, macropain) activator subunit 2 (PA28 beta) | PSME2 | nc |
| phosphofructokinase, platelet | PFKP | nc |
| proteasome (prosome, macropain) subunit, beta type, 9 | PSMB9 | nc |
| proteasome (prosome, macropain) subunit, alpha type, 2 | PSMA2 | nc |
| 2'-5'-oligoadenylate synthetase 2, 69/71kDa | OAS2 | 2 |
| Extracelullar mediators | | |
| pentraxin 3, long | PTX3 | nc |
| apolipoprotein L, 3 | APOL3 | 4.4 |
| insulin-like growth factor binding protein 4 | IGFBP4 | nc |
| apolipoprotein L, 1 | APOL1 | 1.9 |
| platelet-derived growth factor alpha polypeptide | PDGFA | nc |
| endothelin 1 | EDN1 | nc |
| apolipoprotein L, 2 | APOL2 | 1.9 |
| inhibin, beta A | INHBA | nc |
| apolipoprotein L, 6 | APOL6 | 2.1 |
| DNA-binding factors | | |
| HESX homeobox 1 | HESX1 | 3.1 |
| interferon regulatory factor 1 | IRF1 | 1.7 |
| activating transcription factor 3 | ATF3 | 2.9 |
| interferon regulatory factor 7 | IRF7 | 1.6 |

^#^Examined M1 genes are based on the transcriptional profiling work of Martinez et al..^2^ nc, No change.

**Table S9** Comparison of M2 genes expressed in Ad-US31-infected THP1 cells#

| Category and Full Gene Name | Gene Title | Fold Change |
| --- | --- | --- |
| Membrane receptors |  |  |
| transforming growth factor, beta receptor II (70/80kDa) | TGFBR2 | nc |
| histamine receptor H1 | HRH1 | 2.4 |
| toll-like receptor 5 | TLR5 | nc |
| macrophage scavenger receptor 1 | MSR1 | 1.7 |
| chemokine (C-X-C motif) receptor 4 | CXCR4 | nc |
| purinergic receptor P2Y, G-protein coupled, 14 | P2RY14 | nc |
| membrane-spanning 4-domains, subfamily A, member 6A | MS4A6A | nc |
| CD36 molecule (thrombospondin receptor) | CD36 | 1.7 |
| membrane-spanning 4-domains, subfamily A, member 4A | MS4A4A | nc |
| mannose receptor, C type 1 | MRC1 | nc |
| Cytokines and chemokines |  |  |
| insulin-like growth factor 1 (somatomedin C) | IGF1 | 6.7 |
| chemokine (C-C motif) ligand 23 | CCL23 | nc |
| chemokine (C-C motif) ligand 18 (pulmonary and activation-regulated) | CCL18 | nc |
| chemokine (C-C motif) ligand 13 | CCL13 | 6.3 |
| Solute carriers |  |  |
| solute carrier family 4, sodium bicarbonate cotransporter, member 7 | SLC4A7 | nc |
| solute carrier family 38, member 6 | SLC38A6 | 1.6 |
| Enzymes |  |  |
| cathepsin C | CTSC | nc |
| hexosaminidase B (beta polypeptide) | HEXB | nc |
| lipase A, lysosomal acid, cholesterol esterase | LIPA | nc |
| adenosine kinase | ADK | nc |
| histamine N-methyltransferase | HNMT | nc |
| tyrosylprotein sulfotransferase 2 | TPST2 | nc |
| ceramide kinase | CERK | nc |
| heparan sulfate (glucosamine) 3-O-sulfotransferase 2 | HS3ST2 | nc |
| leukotriene A4 hydrolase | LTA4H | nc |
| carbonic anhydrase II | CA2 | nc |
| arachidonate 15-lipoxygenase | ALOX15 | nc |
| heparan sulfate (glucosamine) 3-O-sulfotransferase 1 | HS3ST1 | nc |
| Extracelullar mediators |  |  |
| transforming growth factor, beta-induced, 68kDa | TGFBI | 1.6 |
| selenoprotein P, plasma, 1 | SEPP1 | -3.6 |
| chimerin 2 | CHN2 | -2 |
| fibronectin 1 | FN1 | 2.4 |
| fibrinogen-like 2 | FGL2 | 3.1 |
| DNA-binding factors |  |  |
| growth arrest-specific 7 | GAS7 | 1.8 |
| early growth response 2 | EGR2 | nc |
| v-maf musculoaponeurotic fibrosarcoma oncogene homolog (avian) | MAF | nc |

^#^Examined M2 genes are based on the transcriptional profiling work of Martinez et al..^2^ nc, No change.

**Table S10** Comparison of M1 genes expressed in Ad-US31-infected THP1 derived macrophages#

| Category and Full Gene Name | Gene Title | Fold Change |
| --- | --- | --- |
| Membrane receptors | | |
| chemokine (C-C motif) receptor 7 | CCR7 | 3.4 |
| interleukin 2 receptor, alpha | IL2RA | 1.8 |
| interleukin 15 receptor, alpha | IL15RA | 1.4 |
| interleukin 7 receptor | IL7R | nc |
| Cytokines and chemokines | | |
| chemokine (C-X-C motif) ligand 11 | CXCL11 | 6.4 |
| chemokine (C-X-C motif) ligand 10 | CXCL10 | 9.6 |
| chemokine (C-X-C motif) ligand 9 | CXCL9 | nc |
| tumor necrosis factor | TNF | 1.5 |
| chemokine (C-C motif) ligand 5 | CCL5 | 1.6 |
| interleukin 12B | IL12B | -3.3 |
| interleukin 15 | IL15 | nc |
| chemokine (C-C motif) ligand 20 | CCL20 | 2.2 |
| Apoptosis-related genes | | |
| BCL2-related protein A1 | BCL2A1 | 2.7 |
| Fas (TNF receptor superfamily, member 6) | FAS | 1.5 |
| baculoviral IAP repeat containing 3 | BIRC3 | 1.9 |
| growth arrest and DNA-damage-inducible, gamma | GADD45G | 1.6 |
| Solute carriers | | |
| solute carrier family 7, member 5 | SLC7A5 | nc |
| solute carrier family 2, member 6 | SLC2A6 | nc |
| solute carrier family 31, member 2 | SLC31A2 | nc |
| Enzymes | | |
| phospholipase A1 member A | PLA1A | nc |
| 2'-5'-oligoadenylate synthetase-like | OASL | 6 |
| chitinase 3-like 2 | CHI3L2 | nc |
| hydroxysteroid (11-beta) dehydrogenase 1-like | HSD11B1L | nc |
| adenylate kinase 3 | AK3 | nc |
| sphingosine kinase 1 | SPHK1 | nc |
| 6-phosphofructo-2-kinase/fructose-2,6-biphosphatase 3 | PFKFB3 | nc |
| proteasome (prosome, macropain) activator subunit 2 (PA28 beta) | PSME2 | nc |
| phosphofructokinase, platelet | PFKP | nc |
| proteasome (prosome, macropain) subunit, beta type, 9 | PSMB9 | 1.7 |
| proteasome (prosome, macropain) subunit, alpha type, 2 | PSMA2 | nc |
| 2'-5'-oligoadenylate synthetase 2, 69/71kDa | OAS2 | 5.2 |
| Extracelullar mediators | | |
| pentraxin 3, long | PTX3 | nc |
| apolipoprotein L, 3 | APOL3 | nc |
| insulin-like growth factor binding protein 4 | IGFBP4 | 1.5 |
| apolipoprotein L, 1 | APOL1 | 1.7 |
| platelet-derived growth factor alpha polypeptide | PDGFA | nc |
| endothelin 1 | EDN1 | 2.3 |
| apolipoprotein L, 2 | APOL2 | 1.9 |
| inhibin, beta A | INHBA | nc |
| apolipoprotein L, 6 | APOL6 | 1.6 |
| DNA-binding factors | | |
| HESX homeobox 1 | HESX1 | 1.7 |
| interferon regulatory factor 1 | IRF1 | nc |
| activating transcription factor 3 | ATF3 | 2.2 |
| interferon regulatory factor 7 | IRF7 | 3.4 |

^#^Examined M1 genes are based on the transcriptional profiling work of Martinez et al..^2^ nc, No change.

**Table S11 Comparison of M2 genes expressed in Ad-US31-infected THP1 derived macrophages#**

| Category and Full Gene Name | Gene Title | Fold Change |
| --- | --- | --- |
| Membrane receptors | | |
| transforming growth factor, beta receptor II (70/80kDa) | TGFBR2 | -1.6 |
| histamine receptor H1 | HRH1 | nc |
| toll-like receptor 5 | TLR5 | nc |
| macrophage scavenger receptor 1 | MSR1 | 3.3 |
| chemokine (C-X-C motif) receptor 4 | CXCR4 | nc |
| membrane-spanning 4-domains, subfamily A, member 6A | MS4A6A | 2.1 |
| CD36 molecule (thrombospondin receptor) | CD36 | nc |
| membrane-spanning 4-domains, subfamily A, member 4A | MS4A4A | 2.7 |
| Cytokines and chemokines | | |
| insulin-like growth factor 1 (somatomedin C) | IGF1 | nc |
| chemokine (C-C motif) ligand 23 | CCL23 | -2.9 |
| chemokine (C-C motif) ligand 18 | CCL18 | nc |
| Solute carriers | | |
| solute carrier family 4, sodium bicarbonate cotransporter, member 7 | SLC4A7 | nc |
| solute carrier family 38, member 6 | SLC38A6 | nc |
| Enzymes | | |
| cathepsin C | CTSC | nc |
| hexosaminidase B (beta polypeptide) | HEXB | nc |
| lipase A, lysosomal acid, cholesterol esterase | LIPA | 3.2 |
| adenosine kinase | ADK | nc |
| histamine N-methyltransferase | HNMT | nc |
| tyrosylprotein sulfotransferase 2 | TPST2 | nc |
| ceramide kinase | CERK | nc |
| heparan sulfate (glucosamine) 3-O-sulfotransferase 2 | HS3ST2 | nc |
| leukotriene A4 hydrolase | LTA4H | nc |
| carbonic anhydrase II | CA2 | nc |
| arachidonate 15-lipoxygenase | ALOX15 | 1.5 |
| heparan sulfate (glucosamine) 3-O-sulfotransferase 1 | HS3ST1 | 1.8 |
| Extracelullar mediators | | |
| transforming growth factor, beta-induced, 68kDa | TGFBI | nc |
| selenoprotein P, plasma, 1 | SEPP1 | nc |
| chimerin 2 | CHN2 | nc |
| fibronectin 1 | FN1 | 2 |
| fibrinogen-like 2 | FGL2 | 2.7 |
| DNA-binding factors | | |
| growth arrest-specific 7 | GAS7 | nc |
| early growth response 2 | EGR2 | 1.7 |
| v-maf musculoaponeurotic fibrosarcoma oncogene homolog (avian) | MAF | nc |

^#^Examined M2 genes are based on the transcriptional profiling work of Martinez et al..^2^ nc, No change.

**Table S12** The total of 143 US31-interacting protein candidates

| Number | Protein Name | Gene Bank |
| --- | --- | --- |
| 1 | LDOC1 | NM_012317.2 |
| 2 | RNH1 | BC000677.2 |
| 3 | RUVBL2 | NM_006666.1 |
| 4 | MYCL | NM_005376.3 |
| 5 | PRDX2 | NM_005809.4 |
| 6 | PRRC2B | BC012289.1 |
| 7 | YEATS4 | NM_006530.2 |
| 8 | EEF1A2 | NM_001958.2 |
| 9 | MRFAP1L1 | NM_152301.3 |
| 10 | L3HYPDH | BC012131.1 |
| 11 | RBMS1 | NM_016836.2 |
| 12 | C1orf123 | NM_017887.1 |
| 13 | ASS1 | NM_000050.4 |
| 14 | AMOTL2 | BC011454.1 |
| 15 | DOK1 | NM_001381.2 |
| 16 | ACBD6 | NM_032360.2 |
| 17 | HNRNPD | NM_002138.3 |
| 18 | SEC13 | BC002634.2 |
| 19 | ALDOC | NM_005165.2 |
| 20 | MAGEA8 | NM_005364.3 |
| 21 | C11orf45 | NM_145013.1 |
| 22 | GAPDH | NM_002046.3 |
| 23 | ADH1B | BC033009.2 |
| 24 | AKR1C1 | NM_001353.5 |
| 25 | GPD1L | NM_015141.2 |
| 26 | PYCR2 | NM_013328.2 |
| 27 | PSMA3 | BC029402.1 |
| 28 | SSBP4 | NM_032627.2 |
| 29 | OVOL2 | NM_021220.2 |
| 30 | WDR54 | BC051753.1 |
| 31 | RFX4 | BC028582.2 |
| 32 | GLUL | BC031964.1 |
| 33 | PTGR2 | NM_152444.1 |
| 34 | SHCBP1 | NM_024745.2 |
| 35 | KCNAB2 | NM_172130.1 |
| 36 | MAGEC2 | BC013318.1 |
| 37 | ACOX1 | BC010425.1 |
| 38 | FAM49B | BC016345.1 |
| 39 | ALDH4A1 | BC007581.1 |
| 40 | ADAT3 | NM_138422.1 |
| 41 | ACSBG1 | BC009289.2 |
| 42 | EEF1G | BC013918.1 |
| 43 | HGS | NM_004712.3 |
| 44 | MCCC1 | BC004214.2 |
| 45 | LSM5 | NM_012322 |
| 46 | HNRNPU | BC015782.1 |
| 47 | SF3B4 | NM_005850.3 |
| 48 | GSTA3 | BC020619.1 |
| 49 | MTHFD1 | BC050420.1 |
| 50 | HK1 | BC008730.2 |
| 51 | C1QTNF5 | NM_001278431 |
| 52 | KRTAP20-1 | NM_181615.1 |
| 53 | TKFC | NM_015533 |
| 54 | HLCS | NM_000411.4 |
| 55 | EPS8L1 | BC015763.1 |
| 56 | TGM1 | NM_000359.2 |
| 57 | APPL2 | BC033731.1 |
| 58 | DECR2 | BC011968.1 |
| 59 | FAM104B | NM_138362.1 |
| 60 | PGD | NM_002631.2 |
| 61 | DLG4 | NM_001365.1 |
| 62 | CAMKK2 | BC026060.2 |
| 63 | C1orf94 | BC007637.1 |
| 64 | PSMC3 | NM_002804.3 |
| 65 | PPIA | BC007104.1 |
| 66 | DCTD | BC001286.1 |
| 67 | ACOT7 | BC017365.2 |
| 68 | PKLR | NM_000298.4 |
| 69 | ASAP2 | NM_003887.1 |
| 70 | SRRT | NM_182800.2 |
| 71 | HTRA4 | BC057765.1 |
| 72 | SDCBP | NM_001007067.1 |
| 73 | SDS | BC020750.1 |
| 74 | HSPA2 | BC036107.1 |
| 75 | LOR | BC034690.1 |
| 76 | GAGE4 | NM_001040663 |
| 77 | CTDP1 | NM_004715.4 |
| 78 | IGHG1 | BC019337.1 |
| 79 | NAMPT | BC020691.1 |
| 80 | CRYL1 | NM_015974.2 |
| 81 | SASS6 | BC101026.3 |
| 82 | CPNE4 | NM_130808.1 |
| 83 | TEX33 | NM_178552.2 |
| 84 | TIMP1 | BC007097.1 |
| 85 | FMO5 | BC035687.1 |
| 86 | THYN1 | BC093074.1 |
| 87 | HSP90B1 | BC009195.2 |
| 88 | VWA5A | NM_198315.2 |
| 89 | SMARCC1 | BC113465 |
| 90 | RBM47 | XM_005248109 |
| 91 | GPD1 | NM_005276.2 |
| 92 | DAZ2 | BC113006 |
| 93 | CRYZ | BC070058.1 |
| 94 | USP4 | NM_001394.5 |
| 95 | VASP | BC038224.1 |
| 96 | PDE9A | NM_001001567.1 |
| 97 | RUVBL1 | NM_003707.1 |
| 98 | SSBP1 | BC093054.1 |
| 99 | PLA2G6 | NM_001004426.1 |
| 100 | KDM1A | BC040194.2 |
| 101 | VAT1L | NM_020927.1 |
| 102 | MTERF4 | XM_011510633 |
| 103 | LGALS9 | NM_002308.3 |
| 104 | PHGDH | NM_006623.2 |
| 105 | NUP62CL | BC016327.1 |
| 106 | VWA8 | NM_001009814.1 |
| 107 | TBX10 | NM_005995.4 |
| 108 | NEU4 | NM_080741 |
| 109 | Lin28a | NM_024674.4 |
| 110 | PLEKHM2 | BC068599.1 |
| 111 | SH3BGR | BC006371.2 |
| 112 | PGK1 | NM_000291.2 |
| 113 | IDH1 | BC093020.1 |
| 114 | MASP1 | NM_001031849.1 |
| 115 | ALOXE3 | BC101938.1 |
| 116 | NUDT14 | NM_177533 |
| 117 | CUTA | NM_001014837.1 |
| 118 | FAM131B | BC045611.1 |
| 119 | MARCKSL1 | BC066915.1 |
| 120 | GTF2I | BC004472.2 |
| 121 | IGKC | BC056256.1 |
| 122 | ESM1 | BC011989.1 |
| 123 | EHHADH | NM_001966.2 |
| 124 | MAGEA9 | NM_005365.4 |
| 125 | HACE1 | BC034982 |
| 126 | GDPGP1 | NM_001013657 |
| 127 | ZNF783 | BC131569.1 |
| 128 | PDE1B | BC032226 |
| 129 | KRTAP19-3 | NM_181609 |
| 130 | TCP1 | BC000665 |
| 131 | N4BP1 | NM_153029 |
| 132 | MAGI1 | NM_004742 |
| 133 | ZNF696 | NM_030895 |
| 134 | USP7 | BC019107.2 |
| 135 | TXLNB | AL834248 |
| 136 | NFKB2 | BC002844.2 |
| 137 | ME3 | BC022472 |
| 138 | OGDH | BC004964 |
| 139 | EXOC1 | BC020650 |
| 140 | AP1B1 | CT841508 |
| 141 | XDH | NM_000379 |
| 142 | CCDC117 | CR456461 |
| 143 | HSPA6 | BC035665 |

**Table S13** GO terms enriched in the US31 interactome

| Category | Term | Description | Count | % | PValue |
| --- | --- | --- | --- | --- | --- |
| Biological process | GO:0055114 | oxidation-reduction process | 19 | 13.287 | 0.000 |
|  | GO:0061621 | canonical glycolysis | 5 | 3.497 | 0.000 |
|  | GO:0006096 | glycolytic process | 5 | 3.497 | 0.000 |
|  | GO:0006094 | gluconeogenesis | 5 | 3.497 | 0.000 |
|  | GO:1904874 | positive regulation of telomerase RNA localization to Cajal body | 3 | 2.098 | 0.005 |
|  | GO:0043968 | histone H2A acetylation | 3 | 2.098 | 0.005 |
|  | GO:0032435 | negative regulation of proteasomal ubiquitin-dependent protein catabolic process | 3 | 2.098 | 0.011 |
|  | GO:0046168 | glycerol-3-phosphate catabolic process | 2 | 1.399 | 0.014 |
|  | GO:0009070 | serine family amino acid biosynthetic process | 2 | 1.399 | 0.014 |
|  | GO:0043967 | histone H4 acetylation | 3 | 2.098 | 0.021 |
| Cellular component | GO:0070062 | extracellular exosome | 52 | 36.364 | 0.000 |
|  | GO:0005829 | cytosol | 57 | 39.860 | 0.000 |
|  | GO:0005739 | mitochondrion | 22 | 15.385 | 0.001 |
|  | GO:0005737 | cytoplasm | 56 | 39.161 | 0.001 |
|  | GO:0035267 | NuA4 histone acetyltransferase complex | 3 | 2.098 | 0.007 |
|  | GO:0042995 | cell projection | 4 | 2.797 | 0.017 |
|  | GO:0009331 | glycerol-3-phosphate dehydrogenase complex | 2 | 1.399 | 0.021 |
|  | GO:0072562 | blood microparticle | 5 | 3.497 | 0.024 |
|  | GO:0043209 | myelin sheath | 5 | 3.497 | 0.024 |
|  | GO:0016363 | nuclear matrix | 4 | 2.797 | 0.033 |
| Molecular function | GO:0051287 | NAD binding | 6 | 4.196 | 0.000 |
|  | GO:0005515 | protein binding | 91 | 63.636 | 0.000 |
|  | GO:0051082 | unfolded protein binding | 6 | 4.196 | 0.002 |
|  | GO:0042802 | identical protein binding | 15 | 10.490 | 0.002 |
|  | GO:0016491 | oxidoreductase activity | 7 | 4.895 | 0.005 |
|  | GO:0005524 | ATP binding | 22 | 15.385 | 0.006 |
|  | GO:0050660 | flavin adenine dinucleotide binding | 4 | 2.797 | 0.014 |

**Table S14 HCMV gene expression profiles**

| HCMV Gene | RNA-Seq (this study) | | Allen K. L. Cheung et al. | Felicia D. Goodrum et al. | Cyprian C. Rosset to et al. | |
| --- | --- | --- | --- | --- | --- | --- |
|  | PolyA-seq | Strand specific-seq |  |  | HCMV infected CD14(+) cells | HCMV infected CD34  (+) cells |
| RL2 |  |  | + | + |  |  |
| RL3 |  |  | + | + |  |  |
| RL4 |  |  | + | + |  |  |
| RL5 |  |  | + | + |  |  |
| RL6 |  |  | + | + |  |  |
| RL7 |  |  | + | + |  |  |
| RL11 |  |  | + |  |  |  |
| UL4 |  |  |  | + |  |  |
| UL5 |  |  | + | + |  |  |
| UL13 |  |  |  | + |  |  |
| UL17 |  |  |  | + |  |  |
| UL21 |  |  |  | + |  |  |
| UL23 |  |  | + | + |  |  |
| UL25 |  |  |  | + |  |  |
| UL26 |  |  |  | + |  |  |
| UL28 |  |  |  |  |  | + |
| UL29 | + |  |  |  |  | + |
| UL30 |  |  | + | + |  |  |
| UL32 | + |  |  | + |  |  |
| UL34 | + | + |  | + |  |  |
| UL36 |  | + |  |  |  |  |
| UL37 | + |  |  |  |  | + |
| UL38 |  |  |  | + |  | + |
| UL39 |  |  | + | + |  |  |
| UL40 |  |  |  | + |  |  |
| UL41 |  |  |  | + |  |  |
| UL44 | + | + |  | + | + | + |
| UL50 | + |  |  |  | + | + |
| UL52 |  |  |  |  | + | + |
| UL53 |  |  | + |  |  |  |
| UL56 | + |  | + |  |  |  |
| UL61 |  |  |  | + |  |  |
| UL62 |  |  | + |  |  |  |
| UL63 |  |  |  | + |  |  |
| UL64 |  |  | + | + |  |  |
| UL65 |  |  |  | + |  |  |
| UL66 |  |  | + | + |  |  |
| UL67 |  |  | + | + |  |  |
| UL68 |  |  | + | + |  |  |
| UL69 | + |  |  | + |  |  |
| UL70 |  |  |  | + |  |  |
| UL71 |  |  | + |  |  |  |
| UL72 |  |  |  | + |  |  |
| UL73 |  |  |  | + |  |  |
| UL75 |  |  |  | + |  |  |
| UL76 |  |  |  | + |  |  |
| UL77 |  |  |  | + |  |  |
| UL78 |  |  |  | + |  |  |
| UL79 |  |  |  |  | + | + |
| UL80 |  |  |  | + |  |  |
| UL81 |  |  | + | + |  |  |
| UL82 | + | + |  |  |  |  |
| UL84 | + | + |  | + | + | + |
| UL87 |  |  |  | + | + | + |
| UL89 |  |  | + |  |  |  |
| UL90 |  |  | + |  |  |  |
| UL93 |  |  | + |  |  |  |
| UL95 | + | + |  |  | + | + |
| UL98 |  |  |  | + |  |  |
| UL99 |  |  | + | + |  |  |
| UL105 | + |  |  | + |  |  |
| UL108 |  |  | + | + |  |  |
| UL110 |  |  | + | + |  |  |
| UL111 |  |  | + | + |  |  |
| UL111A | |  | + |  | + | + |
| UL112 | + | + |  |  |  |  |
| UL115 |  |  | + |  |  |  |
| UL117 | + |  |  |  |  |  |
| UL122 |  |  |  | + |  |  |
| UL123 |  | + | + | + |  |  |
| UL124 |  |  |  | + |  |  |
| UL125 |  |  |  | + |  |  |
| UL126A | |  |  |  |  | + |
| UL128 |  |  |  | + |  |  |
| UL131 |  |  |  | + |  |  |
| UL132 |  |  | + | + |  |  |
| UL133 |  |  |  | + |  | + |
| UL135 |  |  |  | + |  | + |
| UL138 |  |  |  | + | + | + |
| UL141 |  |  |  | + |  |  |
| UL145 |  |  |  | + |  |  |
| UL147 |  |  | + |  |  |  |
| UL150 |  |  |  | + |  |  |
| UL153 |  |  | + |  |  |  |
| UL154 |  |  | + |  |  |  |
| US3 | + |  |  |  |  |  |
| US6 |  |  |  | + |  |  |
| US8 |  |  |  | + |  |  |
| US10 |  |  |  | + |  |  |
| US11 |  |  |  | + |  |  |
| US12 |  |  | + | + |  |  |
| US17 |  |  |  |  |  | + |
| US28 |  |  | + | + |  |  |
| US31 |  | + |  |  |  |  |
| US32 |  |  | + | + |  |  |
| US34 |  |  |  | + |  |  |
| TRS1 | + | + |  |  |  |  |
| IRS1 |  | + |  |  |  |  |

^+^ represents detectable

**Supplemental Reference**

1. Cheung AK, Abendroth A, Cunningham AL, Slobedman B. Viral gene expression during the establishment of human cytomegalovirus latent infection in myeloid progenitor cells. *Blood* 2006; **108**: 3691-3699.

2. Martinez FO, Gordon S, Locati M, Mantovani A. Transcriptional profiling of the human monocyte-to-macrophage differentiation and polarization: new molecules and patterns of gene expression. *J Immunol* 2006; **177**: 7303-7311.
