# Supplementary figures and images for: The cytomegalovirus protein US31 induces inflammation through mono-macrophages in systemic lupus erythematosus by promoting NF-κB2 activation
Source: Cell Death Dis. 2018 Jan 24;9(2):104. doi: 10.1038/s41419-017-0122-4 (PMC5833803; doi:10.1038/s41419-017-0122-4)

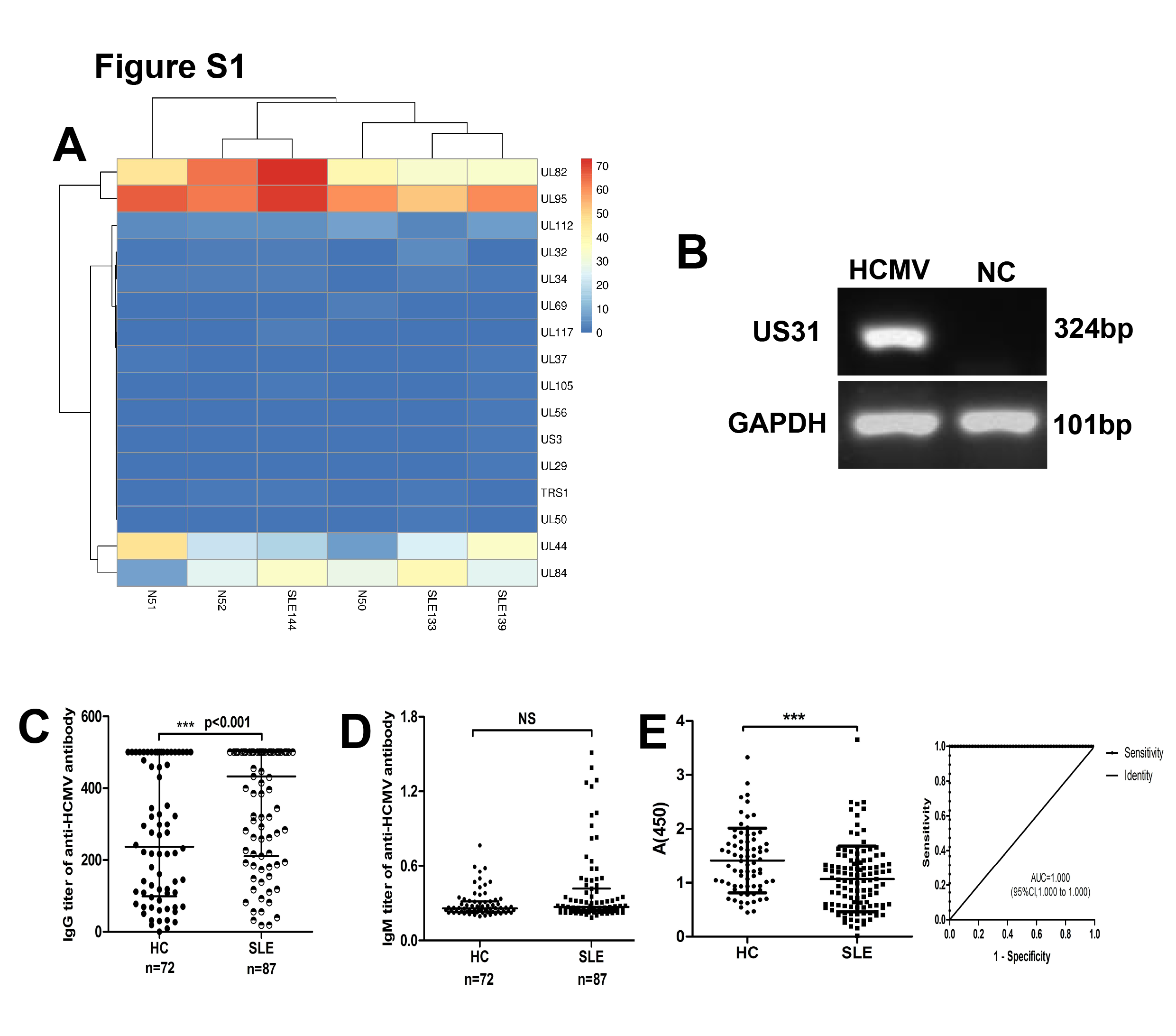

Supplement: Supplementary file 2 — SUPPLEMENTAL Figure S1 [file 41419_2017_122_MOESM2_ESM.tif]

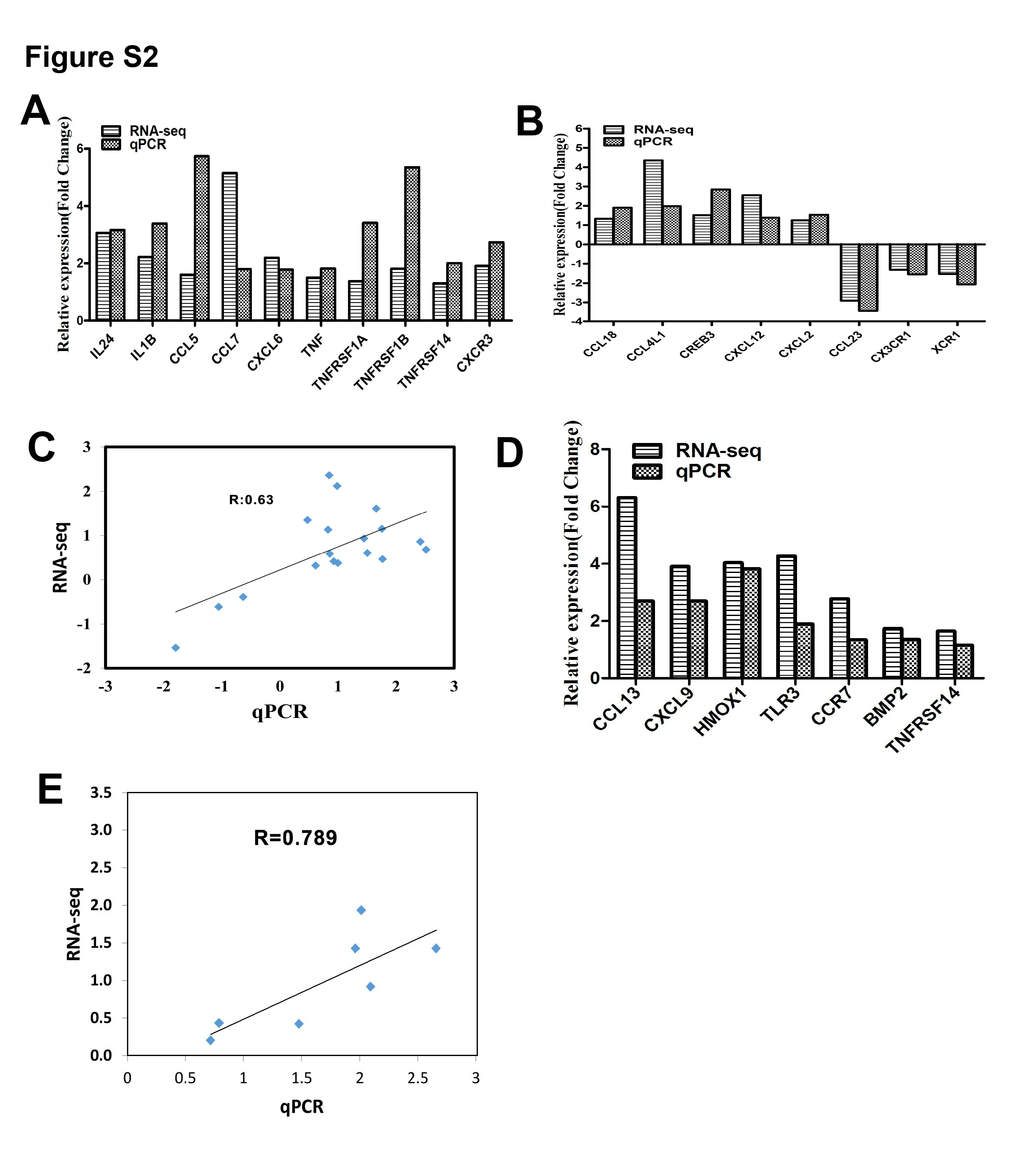

Supplement: Supplementary file 3 — SUPPLEMENTAL Figure S2 [file 41419_2017_122_MOESM3_ESM.tif]

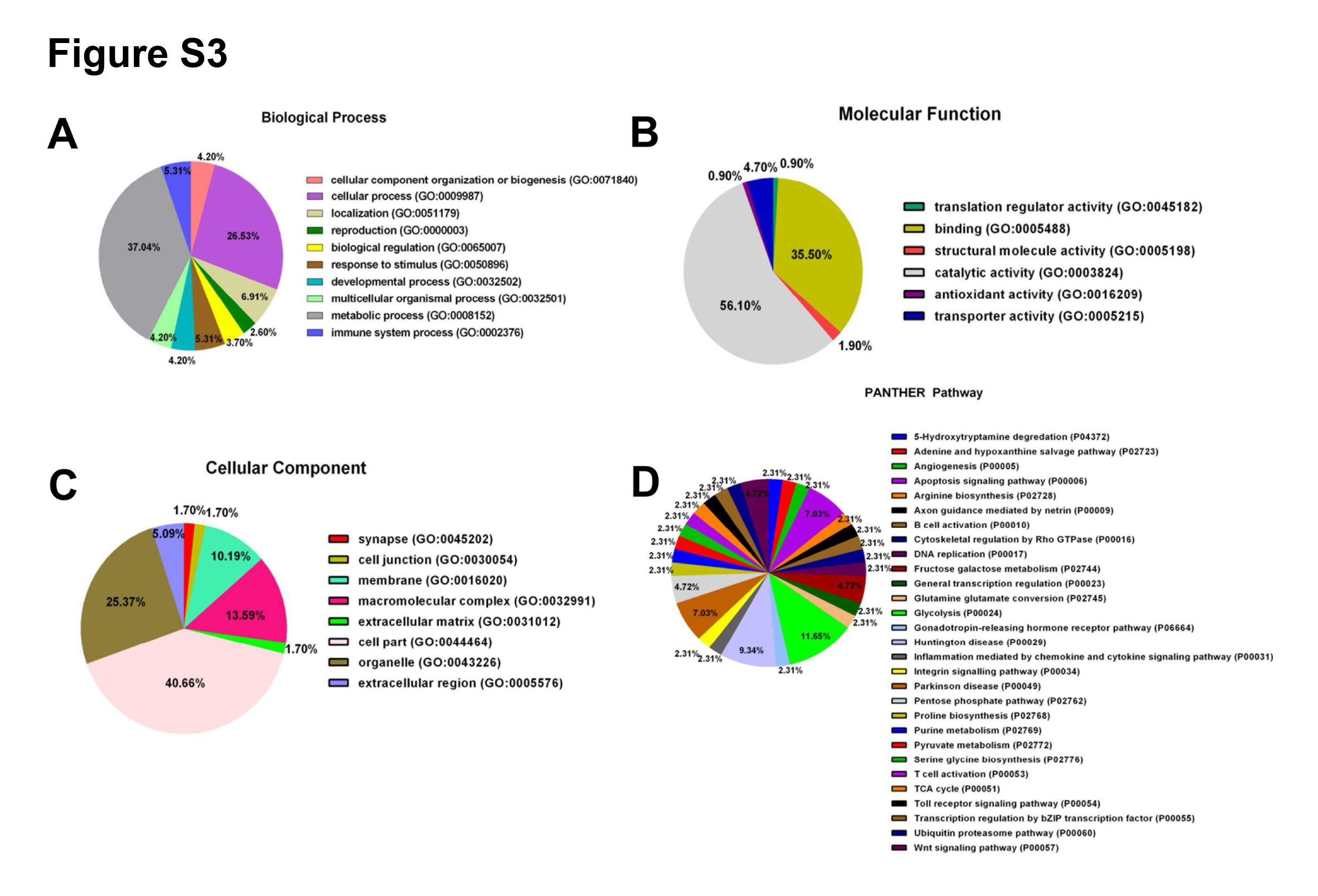

Supplement: Supplementary file 4 — SUPPLEMENTAL Figure S3 [file 41419_2017_122_MOESM4_ESM.tif]

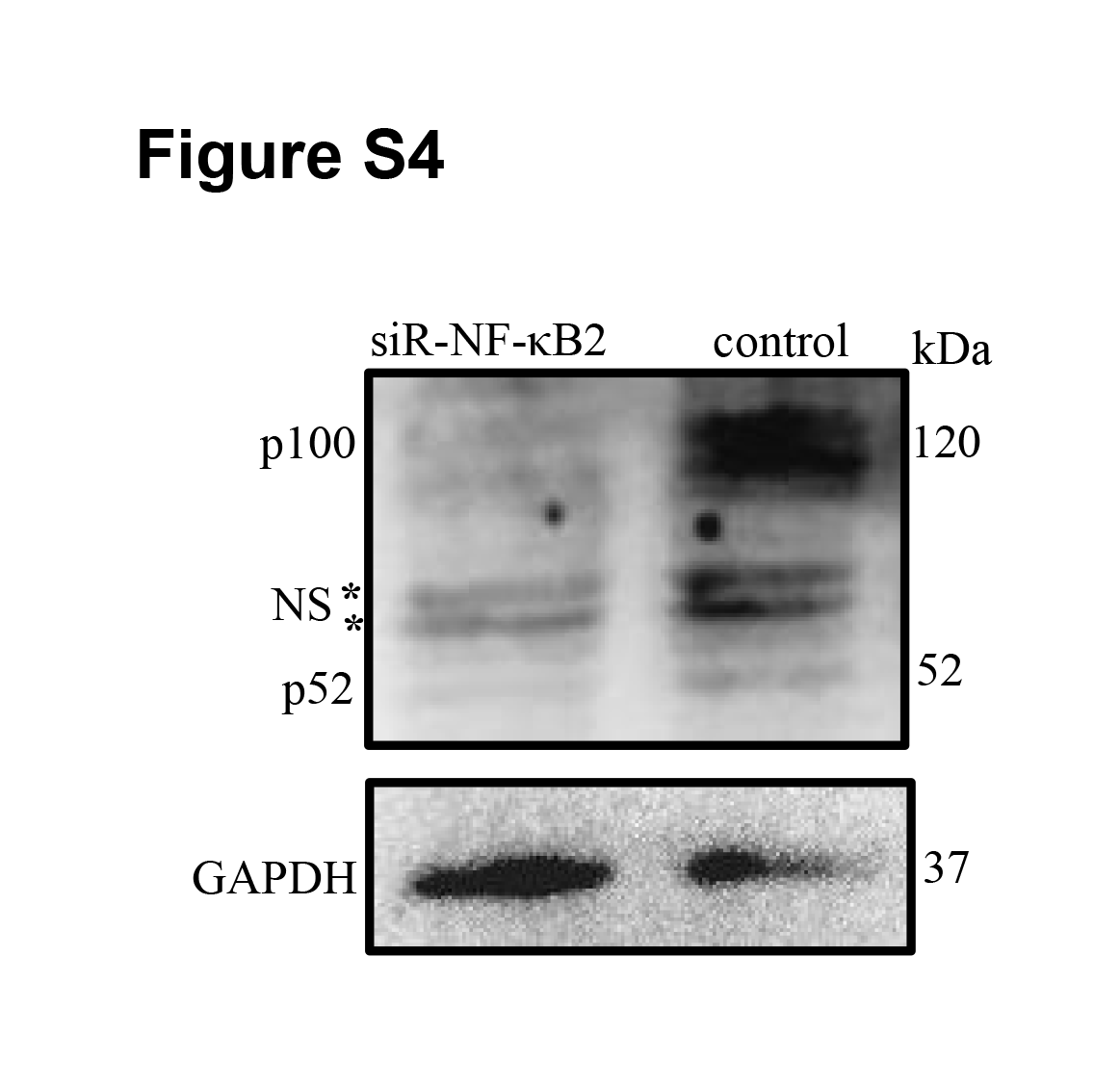

Supplement: Supplementary file 5 — SUPPLEMENTAL Figure S4 [file 41419_2017_122_MOESM5_ESM.tif]

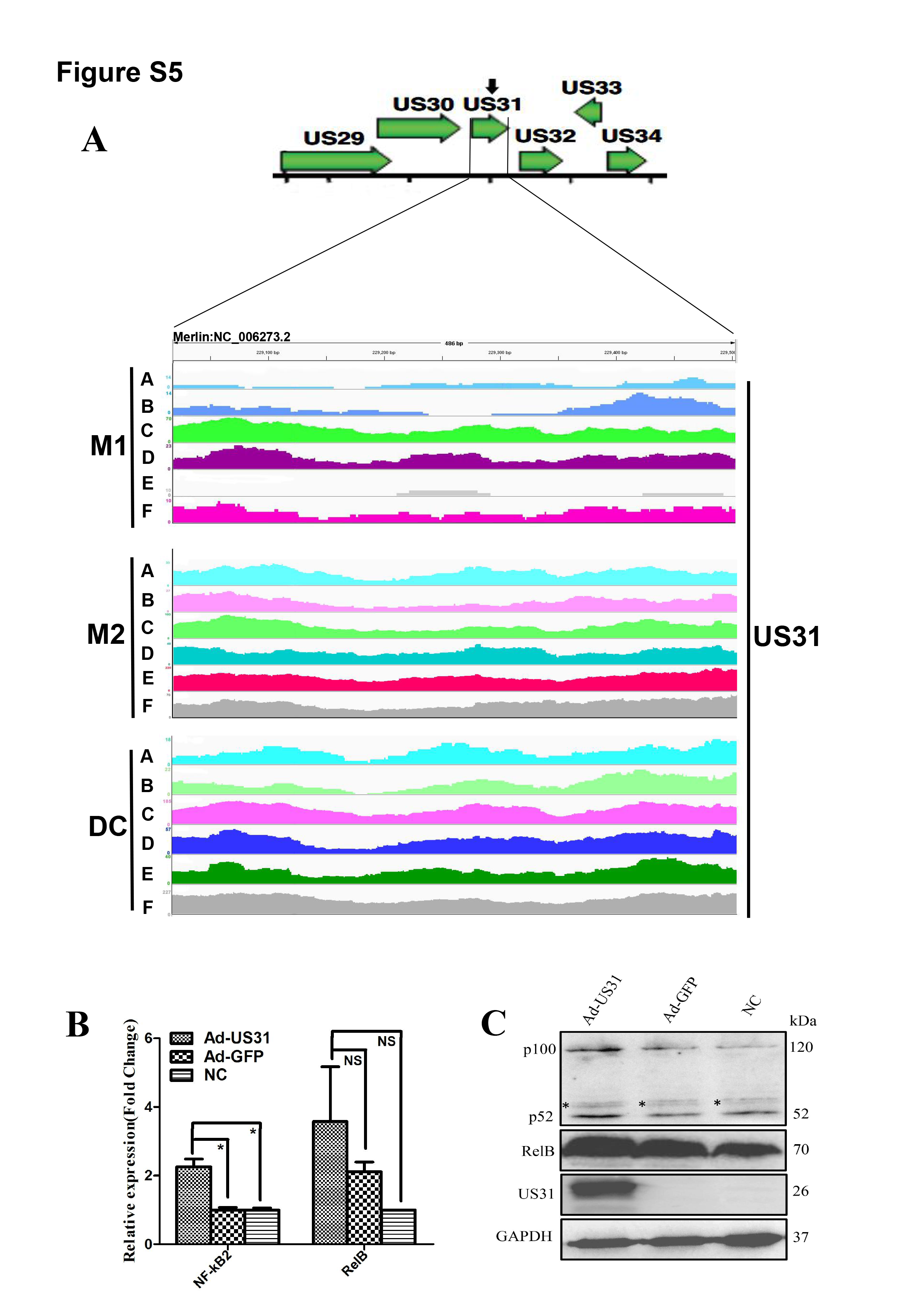

Supplement: Supplementary file 6 — SUPPLEMENTAL Figure S5 [file 41419_2017_122_MOESM6_ESM.tif]
